# An optimized click chemistry method allows visualization of proliferating neuronal progenitors in the mouse brain

**DOI:** 10.1016/j.crmeth.2025.101208

**Published:** 2025-10-21

**Authors:** Fei Zhao, Tomonari Hamaguchi, Ryo Egawa, Atsushi Enomoto, Kinji Ohno

**Affiliations:** 1 Division of Neurogenetics, Center for Neurological Diseases and Cancer, Nagoya University Graduate School of Medicine, Nagoya, Japan; 2 Department of Cell Physiology, Nagoya University Graduate School of Medicine, Nagoya, Japan; 3 Department of Pathology, Nagoya University Graduate School of Medicine, Nagoya, Japan; 4 Graduate School of Nutritional Sciences, Nagoya University of Arts and Sciences, Nisshin, Japan

**Keywords:** copper-catalyzed azide-alkyne cycloaddition,, CuAAC, *in situ* click reaction, tissue clearing, EdU, neuronal progenitors, proliferating cells, cancer cells, embryo

## Abstract

We establish a click reaction-based workflow (tissue clearing coupled with click-chemistry in 3D, C^4^-3D) to visualize 5-ethynyl-2′-deoxyuridine (EdU) in whole mouse brain tissue cleared by CUBIC, iDisco+, and PACT. C^4^-3D was compatible with immunostaining, nuclear staining, and a fluorescent reporter mouse. Machine learning-based identification of EdU-positive nuclear coordinates followed by normalization for the Allen Brain Atlas revealed that proliferating neuronal progenitors were enriched in the subventricular zones (SVZs) and in their migration pathways to the olfactory bulbs and were decreased with aging. C^4^-3D for EdU was also applied to mouse models of cerebral infarction, glioblastoma multiforme, and metastatic brain tumor, as well as to kidney, liver, lung, and embryo in normal mouse. C^4^-3D will enable the exploration of cellular proliferation profiles in 3D. Especially, timed pulse-chase of EdU in normal development, disease progression, and tissue repair coupled with immunostaining will disclose the spatiotemporal generation, migration, and differentiation of newly synthesized cells.

## Highlights

• C^4^-3D allows EdU visualization in whole mouse brain cleared by CUBIC, iDisco+, and PACT
• C^4^-3D is compatible with immunostaining, nuclear staining, and reporter mouse
• 3D mapping reveals enrichment of EdU+ cells in SVZ that declines with aging
• C^4^-3D is applied to disease models and normal organs such as liver and kidneys

## Motivation

Three-dimensional identification of proliferating cells in whole organs remains technically challenging because of incompatibility between DNA synthesis markers and tissue-clearing methods. In particular, antibody-based BrdU detection is not feasible in cleared tissues due to the harsh denaturation steps in tissue clearing. While EdU-based click chemistry offers a promising alternative, its efficient application in 3D imaging in whole organs, especially for high-resolution and region-specific analysis, such as in the subventricular zone in the brain, remains underdeveloped. This study addresses the gap by establishing a robust and versatile protocol that integrates three tissue-clearing methods, click chemistry, and spatial mapping.

## Graphical abstract

**Figure:**
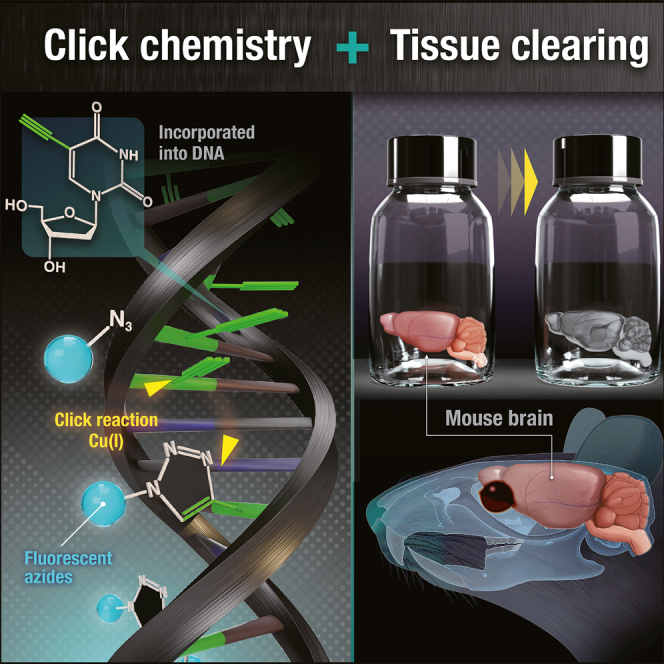

## Teaser

Zhao et al. present an optimized 3D click chemistry method (C^4^-3D) that enables visualization of EdU-labeled proliferating cells in tissue-cleared whole mouse brains. The method is compatible with major tissue-clearing protocols and fluorescent reporters and allows precise mapping of proliferating cells to the Allen Brain Atlas enabling spatiotemporal identification of neurogenesis in development, injury, aging, and disease models.

## Introduction

Analysis of cellular proliferation in organs traditionally depends on reconstruction of a 3D image from serial cryostat sections.^1^^,^^2^ However, the preparation and the visual acquisition of serial cryostat sections of a large sample are extremely laborious with occasional loss of sections, and the reconstruction requires high computational resources.^3^^,^^4^ To address these challenges, optical sectioning techniques such as confocal microscopy and two-photon microscopy have been developed, which effectively increased the imaging depth. However, the imaging depth remains limited to approximately 1 mm in uncleared tissues.^5^ Tissue-clearing techniques effectively overcome the shortcomings of traditional slicing methods and provide several millimeters of imaging depth, while the sample integrity is preserved.^6^ The tissue-clearing techniques have become a powerful tool for morphological studies from whole organisms to subcellular structures. Laborious optimizations in refractive index matching, enhanced hydration, lipid dissolution, hydrogel embedding, and advancement in light sheet fluorescence microscopy (LSFM)^7^ improved the tissue fidelity and facilitated the discernment of immunohistochemical signals and intrinsic autofluorescence.^8^

The assessment of cellular proliferation depends on accurate identification of DNA synthesis. A copper-catalyzed click reaction with fluorescent azide dye enables rapid and specific detection of the alkyne click handle in 5-ethynyl-2′-deoxyuridine (EdU)^9^ incorporated into DNA in tissue sections and cultured cells.^10^ A key advantage of this technique is its bioorthogonality, i.e., it does not interfere with other biological molecules.^11^ This technique visualizes the 2D distribution of newly synthesized DNA carrying EdU in *in vitro*, *ex vivo*, and *in vivo* imaging. An alternative and more commonly used technique in 2D is the incorporation of 5-bromo-2′-deoxyuridine (BrdU) into newly synthesized DNA, and its detection with a specific antibody. However, DNA denaturation for immunostaining BrdU requires either acidification or high temperate, both of which damage cleared tissues and cannot be used in 3D. In contrast to BrdU, EdU detection does not depend on an antibody, which simplifies the detection process and significantly shortens the detection time.^12^^,^^13^ Tissue-clearing with conventional click chemistry has been reported in 500^14^ and 1,500 μm^15^ sections, although the specimens were not analyzed by LSFM. Tissue-clearing by CUBIC with modified click chemistry was also reported for the whole mouse brain.^16^

The Allen Brain Atlas is commonly used to count the number of cells in specific brain regions.^17^^,^^18^^,^^19^^,^^20^^,^^21^ However, proliferation in the brain mostly occurs at the boundaries of anatomical structures, which makes it difficult to map proliferating neuronal progenitors in specific brain regions in the Allen Brain Atlas. For example, the subventricular zone (SVZ) that is enriched in neuronal progenitors constitutes a thin layer on the caudoputamen and is not annotated in the Allen Brain Atlas.

Here we developed a technique (C^4^-3D) to perform the *in situ* click reaction to visualize EdU in whole mouse brain tissue cleared by CUBIC, iDisco+, and PACT along with machine learning-based mapping of EdU-positive nuclei in the Allen Brain Atlas that was additionally annotated for the SVZ and nestin.

## Results

### Optimization of protocols for the *in situ* click reaction to detect EdU signals in whole mouse brain tissue cleared by CUBIC, iDisco+, and PACT

**Figure 1:**
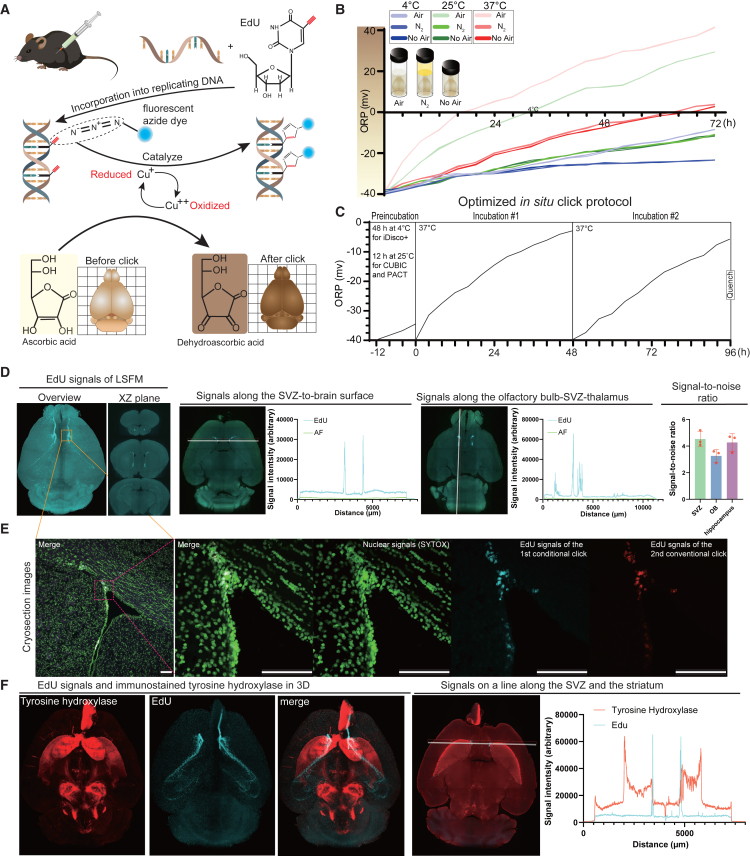
Tissue-clearing coupled with *in situ* click reaction (C^4^-3D) reveals cell proliferation niches in the mouse brain (A) EdU with a click handle (red) is incorporated into replicating DNA. The click handle is catalyzed with a fluorescent azide dye (blue ball) by a reduced copper ion. Ascorbic acid reduces divalent copper to monovalent copper. The oxidized dehydroascorbic acid causes pigmentation, which cannot be cleared by additional tissue clearing (Figure S1). (B) Oxidation-reduction potentials (ORP) of the Cu/SL-AA solution under different temperatures and different equilibrated gases in 72 h. (C) Representative ORP in the reaction mixture of the optimized *in situ* click protocol (Figure 2). In the optimized protocol, the EdU-treated brain was preincubated in 0.1 M SL-AA without CuSO_4_ at 25°C for 12 h (CUBIC and PACT) or at 4°C for 48 h (iDisco+), and then incubated with 0.1 M SL-AA and 0.1 mM CuSO_4_ at 37°C for 48 h twice. (D) (Left) Representative maximum intensity projection (MIP) image stacking the z axis of light sheet fluorescent microscope (LSFM) of the mouse brain at age 2 months visualized by the optimized *in situ* click protocol shown in (C). See Figure S2A for schematic diagram of the experiment. Signal intensities on the indicated white lines on the SVZ-to-brain surface (2nd panel) and the olfactory bulb-SVZ-thalamus (3rd panel) are indicated. AF, autofluorescence. Signal-to-noise ratios (SNRs) were calculated for the SVZ, olfactory bulb (OB), and hippocampus and are shown in the rightmost bar graph (mean and SD, *n* = 3 mice). SNR was defined as the mean signal intensity divided by the mean background intensity. (E) EdU (blue)-stained whole brain in (D) was cryosectioned at the level of the orange dotted rectangle. The slice was stained by SYTOX nuclear dye (green) and an additional conventional click reaction (red). See Figure S2B for schematic diagram of the experiment. Each experimental group included three independent brain samples (*n* = 3 mice), and a representative slice is shown. Scale bars: 100 μm. (F) C^4^-3D is compatible with immunostaining. The whole mouse brain subjected to *in situ* click reaction (blue) was immunostained for tyrosine hydroxylase (red) in 3D.

As shown in Figure 1A, EdU incorporated into DNA in S-phase cells was added with a fluorescent azide dye by monovalent copper in click chemistry. We first used the original CuSO_4_/sodium L-ascorbate (Cu/sodium L-ascorbic acid [SL-AA]) solution containing 1 mM CuSO_4_, 0.1 M SL-AA, and 30 ng/mL of an azide dye (AZDye 568 Azide Plus or AZDye 647 Azide Plus) in phosphate buffered saline (PBS) that has been developed for the click reaction in 2D.^22^ However, the Cu/SL-AA solution could not perform the click reaction deep inside the brain, because both copper and SL-AA were oxidized over time at any temperature and even when oxygen was excluded (Figure 1B). Dehydroascorbic acid, an oxidized form of ascorbic acid with a yellow color,^23^^,^^24^ caused tissue pigmentation and enhanced autofluorescence, which could not be cleared either by additional CUBIC, iDisco+, or PACT (Figure S1).

**Figure 2:**
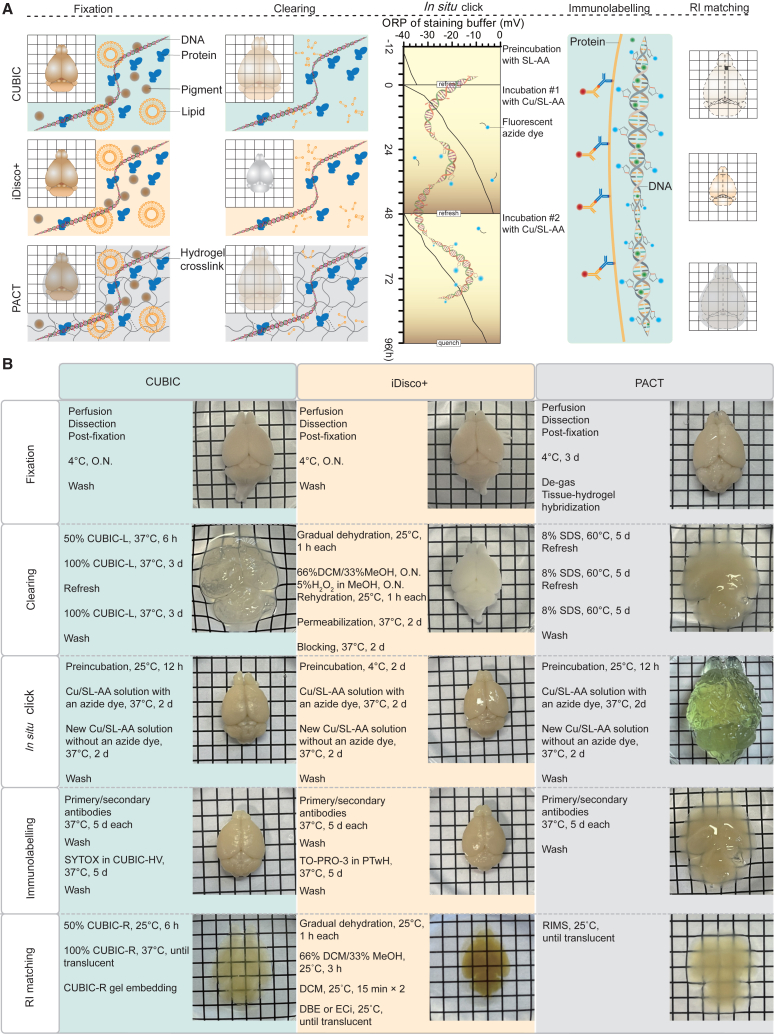
Optimized protocols of C^4^-3D for CUBIC, iDisco+, and PACT (A and B) Schematic presentations (A) and the detailed protocols (B) of C^4^-3D for CUBIC, iDisco+, and PACT. Brain samples were tissue cleared according to the standard protocols of CUBIC, iDisco+, and PACT. For *in situ* click reaction, samples were preincubated with a reduced SL-AA solution for 12 h at 25°C for CUBIC and PACT and at 4°C for iDisco+. Thereafter, the samples were incubated with a reduced click solution (Cu/SL-AA) for 48 h twice at 37°C. O.N., overnight; CUBIC-L, clear unobstructed brain/body imaging cocktails and computational analysis-L for lipid removal; CUBIC-R, CUBIC-R for refractive index matching; CUBIC-HV, CUBIC-HistoVision; DCM, dichloromethane; MeOH, methanol; PTwH, PBS with Tween 20 and heparin; DBE, dibenzyl ether; Eci, ethanol-chloroform-iodine; SDS, sodium dodecyl sulfate; and RIMS, refractive index matching solution.

We optimized the conditions by monitoring the *in situ* click reaction of whole mouse brain tissue cleared by CUBIC in two tracks (Figure S2). The first optimization track was to monitor EdU signals and autofluorescence on a line drawn on a computationally sliced image of whole mouse brain that was stained by a conditional *in situ* click reaction of whole mouse brain (Figure S2A). The second optimization track was to detect click handles of EdU deep in the brain that remained unreacted in the 1st conditional *in situ* click reaction. After the 1st conditional *in situ* click reaction, the brain was cryosectioned and the section was subjected to the 2nd conventional click reaction (Figure S2B). We first tried to keep copper in a reduced form for four days by adding tert-Butylimino-tri(pyrrolidino)phosphorane (BTTP) (Figure S3C) and L-histidine (Figure S3D), but found that they rather increased autofluorescence. Instead, we found that the addition of a new Cu/SL-AA solution in 48 h achieved high signal-to-noise ratios (Figures 1C–1E). Pervasive background signals in 3D (Figure 1D) compared to those in 2D (Figure 1E) were because multiple planes were compressed into a single plane along the *z* axis in 3D, whereas a section with the strongest SVZ signals was taken in 2D. We also found that preincubation of whole mouse brain with SL-AA and an azide dye in the absence of copper ions was essential to make the *in situ* click reaction take place under a reduced condition, because the omission of the SL-AA preincubation step generated weak and uneven EdU labeling (Figure S1E). We next performed the *in situ* click reaction at 4°C, 25°C, and 37°C, and found that 37°C gave rise to the best signal-to-noise ratio (Figure S4). We also performed *in situ* click reaction without EdU injection to show that higher cortical background compared to the central background in Figure S4 was due to absorption of the excitation light of the light sheet fluorescent microscopy on the sample surface (Figure S1D). The optimized C^4^-3D protocols for whole mouse brain tissue cleared by CUBIC, iDisco+, and PACT are summarized in Figure 2. Representative C^4^-3D images for EdU with iDisco+ and PACT are shown in Figures S5A–S5D. We also confirmed that C^4^-3D of whole brain was compatible with immunostaining for tyrosine hydroxylase (Figure 1F; Video S1) and a proliferation marker Ki-67 (Figures 5A–5E), as well as with chemical nuclear staining by SYTOX for CUBIC and TO-PRO-3 for iDisco+ (Figure 5I). The C^4^-3D mouse brain was also compatible with a fluorescent reporter mouse brain expressing nestin-tdTomato (Figures S6A–S6C).

Video S1. Representative image of tyrosine hydroxylase (red) and EdU (blue) of the mouse brain tissue cleared by CUBIC, related to Figure 1 The video was generated by Imaris software.

### Mapping of EdU signals to the Allen Brain Atlas for annotating the brain regions

**Figure 3:**
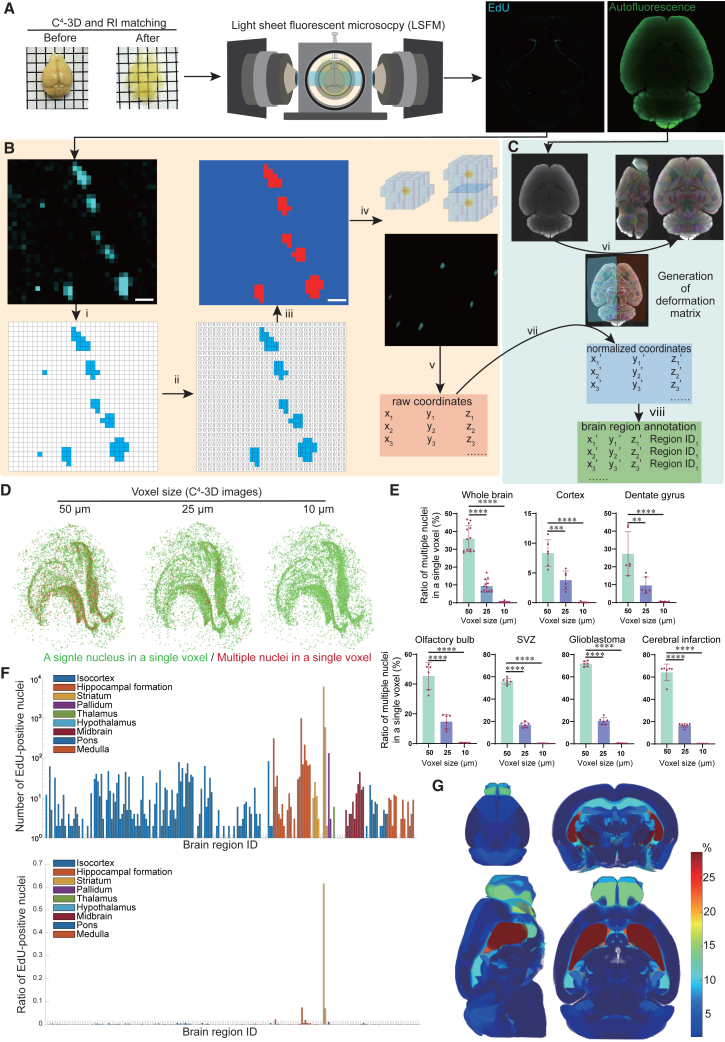
Acquisition of EdU signals and machine learning-based coordinate analysis (A) Workflow of C^4^-3D and image acquisition with light sheet fluorescence microscopy (LSFM). RI, refractive index. (B) Noise elimination and identification of the signal coordinates by Labkit. Scale bars: 10 μm, (i) Background subtraction of captured images. (ii) Elimination of noises with Labkit^25^ for Fiji.^26^ (iii) Visual confirmation of the processed images. (iv)Identification of the center of the nucleus (yellow) by 3D Suite Segmentation tool^27^ for Fiji^26^ and its visual confirmation. (v) Collation of the raw nuclear coordinates in the original images. (C) Normalization of the nuclear coordinates to the Allen Brain Altas, and their annotations. (vi) Alignment of the sample’s autofluorescence images with the Allen Brain Atlas to generate the deformation matrix to convert to the reference atlas using antsRegistration.^28^ (vii) Application of the deformation matrix to the raw nuclear coordinates in (B) to obtain normalized nuclear coordinates. (viii) Annotation of the brain region (Region ID) to each normalized nuclear coordinate. (D) Inclusion of multiple EdU-positive nuclei in a single voxel at different voxel sizes of the Allen Brain Atlas. Green and red dots indicate isolated and multiple EdU-positive nuclei in a single voxel, respectively. (E) The ratios of multiple nuclei mapped to a single voxel in three different voxel sizes in different brain regions of the Allen Brain Atlas, glioblastoma, and cerebral infarction. Note that the ratios are high with high voxel sizes in the SVZ, glioblastoma, and cerebral infarction, where positive signals are congested. Mean and SD are indicated (*n* = 6 mice). ∗∗*p* < 0.01, ∗∗∗*p* < 0.005, and ∗∗∗∗*p* < 0.001 by one-way ANOVA followed by Tukey’s posthoc test. (F) The number (top) and the ratio (bottom) of EdU-positive nuclei in each brain region in the Allen Brain Atlas. Values of a representative mouse brain are indicated. (G) 3D heatmap showing the ratio of EdU-positive nuclei in each brain region.

The workflow of our analysis is summarized in Figures 3A–3C. The EdU and autofluorescent signals were captured in 3D using LSFM (Figure 3A). Then, the signals and noises were differentiated by Labkit^25^ for Fiji,^26^ and the centers of EdU-positive nuclei were identified along with their coordinates by 3D Suite Segmentation tool^27^ for Fiji^26^ (Figure 3B). Validation of the discrimination of signals and noises showed that the accuracies were 0.988–1.000 in five brain regions (Table S1). Similarly, validation of the identification of the number of nuclei in a cluster of EdU signals showed that the accuracies were 0.878–0.927 in five brain regions (Table S2 and Video S2). To correct for brain deformity and sample-to-sample variability, the nuclear coordinates of the captured 3D images were normalized to those of the Allen Brain Atlas^17^^,^^18^^,^^19^^,^^20^^,^^21^ using antsRegistration^28^ (Figure 3C).

**Figure 4:**
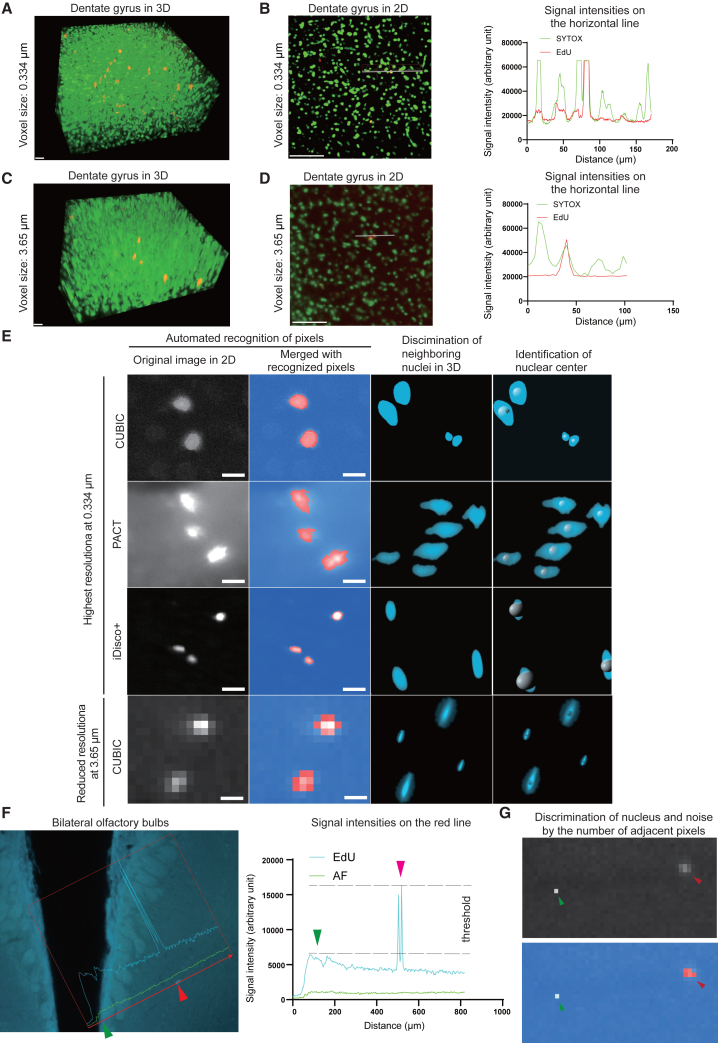
Reduction of the spatial resolution from 0.334 to 3.65 μm without compromising automated identification of each nucleus (A and C) Representative EdU signals (red) and SYTOX signals (green) of the dentate gyrus in 3D at the highest resolution of 0.334 μm (A) and the minimum acceptable resolution of 3.65 μm (C) with LSFM. Scale bars: 100 μm. (B and D) 2D-sliced images of (A and C), respectively. Signals for EdU (red) and SYTOX (green) on the horizontal line show a precise overlap of newly synthesized EdU-positive DNA and SYTOX-positive nucleus. Scale bars: 100 μm. (E) Two left columns: representative EdU signals (white) in a 2D-slice image and their automated recognition (red) by Labkit at different spatial resolutions. Two right columns: Recognized EdU signals in 3D (red) and automated identification of the nuclear centers (balls) by Labkit. The 10-μm balls indicating the nuclear centers look huge for iDisco+, because iDisco+ shrank the brain. Note that nuclear centers could be individually identified even at 3.65 μm resolution. Scale bars: 100 μm. (F) Two adjacent nuclei (EdU signals) in the OB in a 2D-sliced image could be distinctly recognized at 3.65 μm resolution. (G) Representative discrimination of a nucleus and a noise at 3.65 μm resolution. Three or more pixels on the right (red arrowhead) were recognized as a single nucleus, whereas a single pixel on the left (green arrowhead) was recognized as a noise by Labkit.

Video S2. Representative automated identification of nuclear centers (red) from EdU signals (white) in the cortex, subventricular zone, dentate gyrus, glioblastoma, and stroke regions of the mouse brain tissue cleared by CUBIC, related to Figure 4

We initially captured the signals of whole mouse brain in 3D at the highest resolution of 0.334 μm (Figure 4A). We confirmed that EdU signals precisely overlapped with the SYTOX nuclear signals at this resolution in 2D sliced images (Figure 4B). However, the file size became ∼1 terabyte (TB) per channel × 2 channels. We first observed that three or more adjacent voxels are required to be identified as a distinct nucleus in the 3D Suite Segmentation tool. While applying the three-voxel rule and keeping each nucleus separately recognizable, we gradually reduced the spatial resolution to 3.65 μm. Even at this resolution, EdU signals accurately overlapped with the SYTOX nuclear signals in 2D sliced images (Figures 4C and 4D). At this resolution, the file size was reduced to ∼40 gigabytes (GB) per channel × 2 channels. Representative identifications of the centers of the nuclei at high and low resolutions are indicated in Figure 4E. Similarly, representative distinction of two adjacent nuclei at 3.65 μm resolution is indicated in Figure 4F. Additionally, representative distinction of signals and noises at 3.65 μm resolution is indicated in Figure 4G.

The Allen Brain Atlas has three versions with voxel sizes of 50, 25, and 10 μm. We examined whether two or more nuclei were mapped to an identical voxel in the three versions of the Allen Brain Atlas, and found that the voxel size of 10 μm is required to distinctively differentiate two adjacent nuclei (Figures 3D and 3E). Similarly, the voxel size of 10 μm is required to avoid misclassification of brain regions (Figures S5E–S5G). However, when 10% or less overlapping nuclei are acceptable, even 50 μm resolution could be applied for the brain cortex, where EdU-positive nuclei were not enriched (Figure 3E). In this communication, the voxel size of 10 μm was used in the following analysis.

As each voxel in the Allen Brain Atlas has a brain region ID, the normalized nuclear coordinates could be mapped to specific brain regions. This enabled the calculations of the number and the ratio of EdU-positive nuclei in each brain region and revealed that EdU-positive nuclei were enriched in the striatum (Figures 3F and 3G).

### Manual annotation of the SVZ in the Allen Brain Atlas and enrichment of EdU signals in the SVZ

Neuronal progenitors are enriched in the SVZ in the caudoputamen subregion of the striatum. However, the SVZ is not annotated in the Allen Brain Atlas. This omission is likely because the SVZ consists only of a few cell layers facing the lateral ventricles. In the lack of annotated SVZ, EdU-signals are mapped to the caudoputamen subregion in the striatum, which constitutes a very large brain region. We thus manually annotated the SVZ area on the Allen Brain Atlas (Figure 6B and Data S1). We indeed found that EdU signals were markedly enriched in the SVZ (Figures 6D and 6F).

### Comparison of EdU and Ki-67 signals

**Figure 5:**
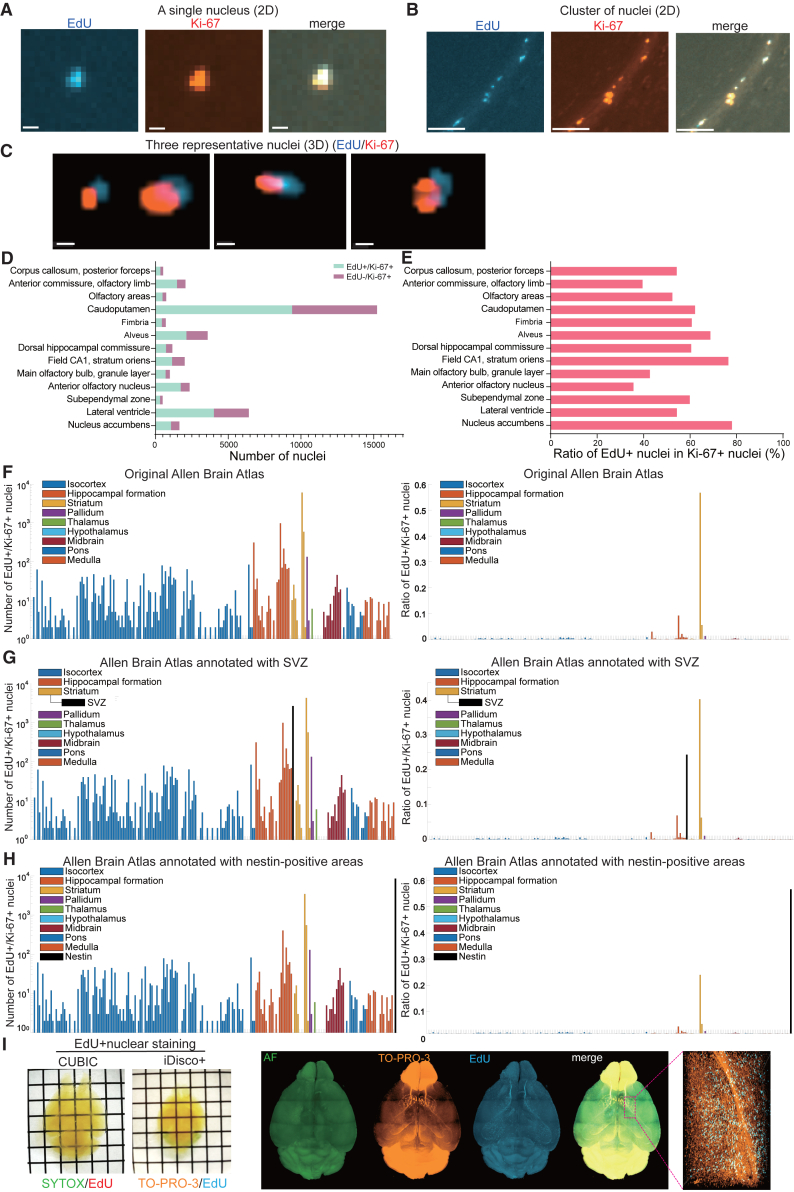
C^4^-3D combined with immunostaining for Ki-67 and chemical nuclear staining (A and B) Representative 2D sliced images of a single nucleus (A) and a cluster of nuclei (B) that are positive for both EdU (blue) and Ki-67 (red). Scale bars: 10 µm (A) and 100 µm (B). (C) Three representative 3D images of nuclei that are positive for both EdU (blue) and Ki67 (red). Note that different absorptions and scattering of the laser beams with different wave lengths^30^^,^^31^ caused marginally different coordinates for EdU and Ki-67 signals, but these signals should have originated from an identical nucleus. Distances less than 5 μm were defined to be colocalized in our analysis. Scale bars: 10 μm. (D) The numbers of EdU+/Ki-67+ nuclei (S phase) and EdU-/Ki-67+ nuclei (late G1, G2, or M phase) in each brain region. Note that all EdU+ nuclei were positive for Ki-67. (E) The ratio of EdU+ nuclei in Ki-67+ nuclei in each brain region. (F–H) The number (left) and the ratio (right) of EdU+/Ki-67+ nuclei in each brain region of the original (F), SVZ-annotated (G), and nestin-annotated (H) Allen Brain Atlas. The SVZ was exclusively delineated from the indicated caudoputamen in three brain regions. The segmentation of SVZ split the number of EdU+ nuclei into the remaining caudoputamen and the SVZ. (D, E, F, G, and H) Representative values in a single sample are indicated. (I) Representative images of double staining of EdU and the nucleus either by SYTOX or TO-PRO-3. An auto-fluorescence (AF) image is also captured and merged on the right.

Simultaneous visualization of EdU by C^4^-3D and Ki-67 by immunostaining (Video S3) showed that EdU-positive nuclei were all positive for Ki-67 but not vice versa (Figures 5A–5C). This is in accordance with the notion that EdU is incorporated in S phase, whereas Ki-67 is expressed in late G1, S, G2, and M phases.^29^ Counting the numbers of EdU+/Ki-67+ and EdU−/Ki-67+ nuclei showed that these nuclei were enriched in the caudoputamen (Figure 5D), but the ratios of EdU+ nuclei in Ki-67+ nuclei were similar across the brain regions (Figure 5E). Similar to EdU+ nuclei stated previously (Figure 3F), EdU+/Ki-67+ nuclei were also enriched in the striatum (Figure 5F). When the SVZ was annotated, EdU+/Ki-67+ nuclei were enriched in the SVZ (Figure 5G).

**Figure 6:**
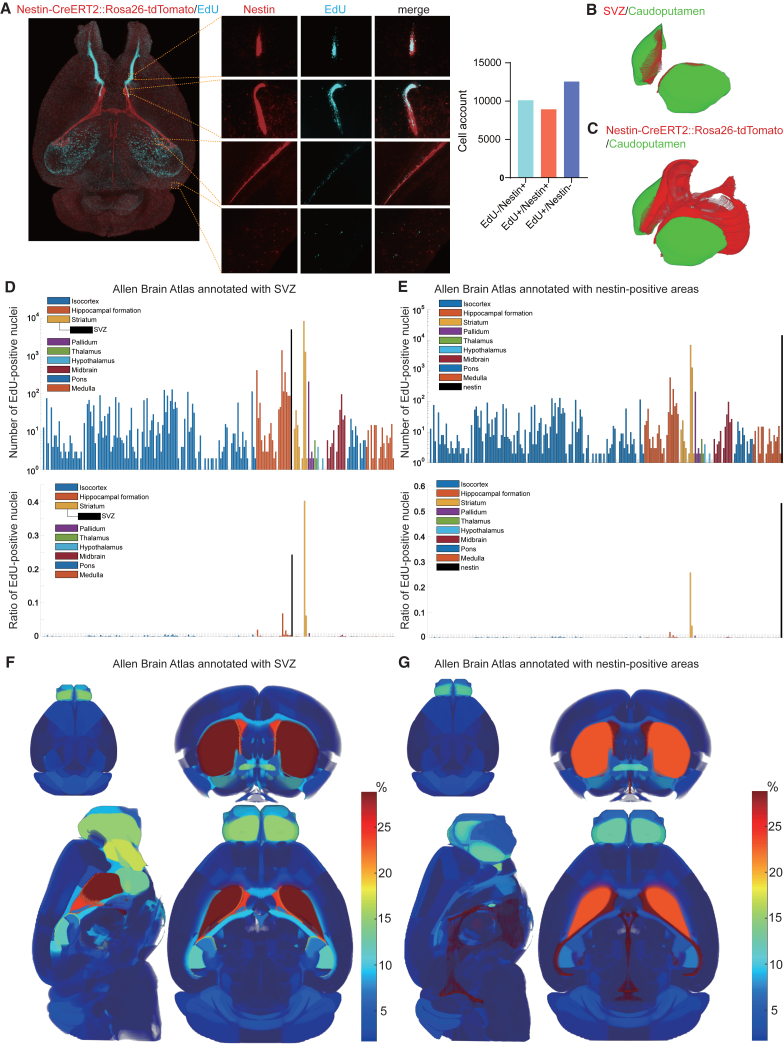
Registration of proliferative functional areas describing neurogenesis patterns (A) Representative 3D image of nestin (red) and EdU (blue) signals in the mouse brain at age 3 weeks. Note that EdU signals in the hippocampus make bundle-like structures. (B) Manually annotated SVZ voxels (red) on the caudoputamen subregion (green) in the striatum of the Allen Brain Atlas. (C) Nestin-positive voxels (red) and the caudoputamen subregion (green) in the striatum of the Allen Brain Atlas. (D) The number (upper panel) and the ratio (lower panel) of EdU-positive nuclei in each brain region of the SVZ-annotated Allen Brain Atlas. The SVZ was not excluded from the striatum and was recognized as a part of the striatum. Values of a representative mouse brain are indicated. (E) The number (top) and the ratio (bottom) of EdU-positive nuclei in each brain region of the nestin-annotated Allen Brain Atlas. In contrast to the SVZ, nestin-positive voxels were excluded from each brain region and were annotated as nestin-positive areas. Values of a representative mouse brain are indicated. (F and G) 3D heatmaps showing the ratio of EdU-positive nuclei in each brain region of the SVZ-annotated (F) and nestin-annotated (G) Allen Brain Atlases.

Video S3. Representative 3D image of EdU (blue) and Ki-67 (yellow) signals in the mouse brain tissue cleared by iDisco+, related to Figure 6 Green signals represent autofluorescence. After 15 s, Ki-67+/EdU+ nuclei are marked in green, and Ki-67+/EdU− nuclei are marked in red.

### Comparison of EdU and nestin signals

As EdU can be taken up even by non-neuronal dividing cells, we next identified neuronal progenitors expressing nestin, an intermediate filament protein, in Nestin-CreERT2::Rosa26-tdTomato reporter mice.^32^^,^^33^ We found that proliferating neuronal progenitors that were positive for both EdU and nestin were enriched in the SVZs and their migratory flows to the olfactory bulbs (Figures 6A, 6C, and S6E). We also found that nestin expression was gradually decreased in the course of migration to the olfactory bulbs. We determined the coordinates of nestin-positive signals and annotated them in the Allen Brain Atlas (Data S2). We found that nestin-positive cells were clustered in specific brain regions, especially at the peripheries of the caudoputamen (Video S4). As expected, EdU signals were enriched in the nestin-positive areas in the Allen Brain Altas (Figures 6E, 6G, and S6C). Similarly, EdU+/Ki-67+ nuclei were enriched in the nestin-positive areas (Figure 5H).

**Figure 7:**
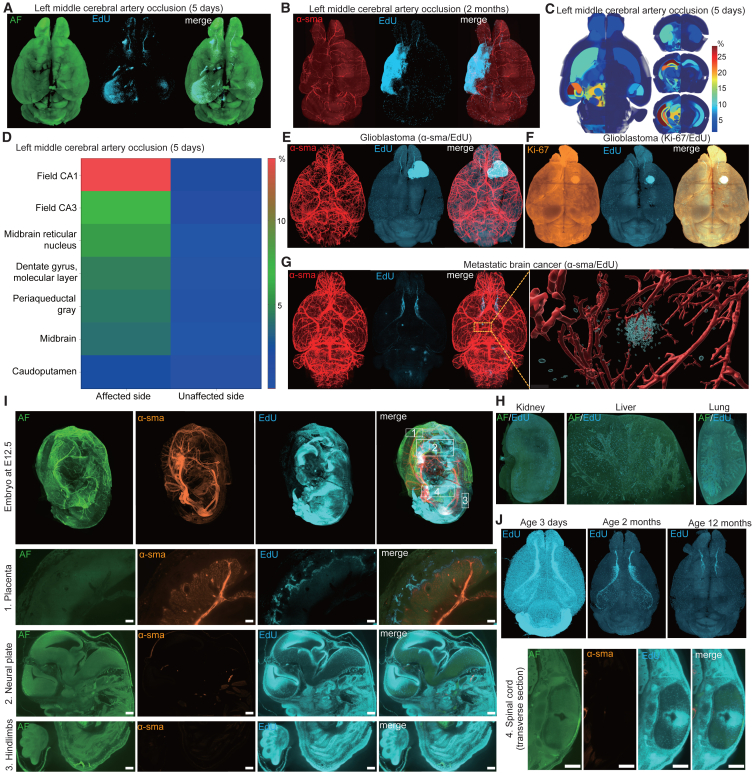
Applications of C^4^-3D to various disease models, as well as to the kidney, liver, lung, embryo, and aging brains in normal mice (A) Representative EdU signals of the mouse brain in 3D at five days after the left middle cerebral artery occlusion (MCAO). AF, autofluorescence. (B) Representative Edu signals and α-smooth muscle action (α-sma) for blood vessels of the mouse brain in 3D at two months after MCAO. (C) Representative horizontal and coronal planes of 3D heatmap showing the ratio of EdU-positive nuclei at five days after MCAO. (D) Heatmap showing the ratio of EdU-positive nuclei in different brain regions at the unaffected and affected sides at five days after MCAO. (E and F) Representative signals of α-sma/EdU (E) and Ki-67/EdU (F) of mouse brains in 3D at four weeks after xenograft transplantation of LN-229 glioblastoma cells. Note that the sizes of glioblastoma were different in the two indicated mice. (G) Representative signals of α-sma/EdU of a mouse brain in 3D at two weeks after intravenous administration of mouse lung cancer cell line, LLC. (H) EdU (blue), α-sma (orange), and autofluorescence (AF, green) of a mouse embryo at E12.5. Four regions of the mouse embryo are enlarged in different panels. (I) EdU (blue) in neonatal, adult, and aged mouse brains. Scale bars: 100 μm. (J) EdU (blue) and autofluorescence (AF, green) of the mouse kidney, liver, and lung at age 8 weeks.

Video S4. Representative 3D image of EdU (blue) and nestin (red) signals in the mouse brain tissue cleared by CUBIC, related to Figure 7 Green signals represent autofluorescence.

### Application of C^4^-3D to mouse models of cerebral infarction, xenotransplanted GBM, and metastatic brain tumor, as well as to the kidney, the liver, the lung, an embryo, and aging brains, to visualize proliferating cells

After brain injury, dormant endogenous neural stem cells become active and participate in the brain repair process. We induced cerebral infarction by occluding the left middle cerebral artery in normal mice at age 8 weeks, and EdU were visualized by C^4^-3D (Figures 7A, 7B, and S6H–S6K; Video S5). Analysis of cell proliferations in each hemisphere showed that proliferating cells were enriched in the affected hemisphere, especially in CA1 in the affected hippocampus in the subacute phase (Figures 5C and 5D). In contrast, in the recovery phase, EdU signals were enriched in atrophic brain area where gliosis was likely to be induced (Figure 7D), as has been previously reported using BrdU in brain injury models including cerebral infarction.^34^^,^^35^^,^^36^

Video S5. Representative 3D image of EdU (blue) and α-smooth muscle actin (α-SMA, red) in the mouse brain 2 months after occlusion of the left middle cerebral artery, related to Figure 8 Note that EdU signals extend from the SVZ to the cortex, which is consistent with an established notion.

We also generated mouse models of xenotransplanted glioblastoma multiforme (GBM) and metastatic brain tumor. The models were subjected to C^4^-3D to visualize EdU, Ki-67, and α-sma, a marker for blood vessels (Figures 7E–7G, S6J, and S6K). As expected, GBM was enriched in EdU and Ki-67 signals, and the arteries entering GBM were twisted (Figures 7E, 7F, and S6J). In addition, the peripheries of GBM were more enriched in EdU and Ki-67 than its center, which was likely to represent insufficient blood supply in the center of GBM (Figure S6K). In contrast, metastatic brain tumor originating from Lewis lung carcinoma (LLC) mouse lung cancer cells were not as congested as GBM, but made a distinct cluster of cells involving blood vessels (Figure 7G).

Furthermore, we applied C^4^-3D to the kidney, the liver, and the lung in normal mouse at age 8 weeks (Figure 7H; Videos S6, S7, and S8), as well as to a mouse embryo at E12.5 (Figure 7I). We followed the same protocols including the incubation times for these organs and the embryo. For these organs and the embryo, the development of reference atlases with regional indices will enable the analysis of region-specific proliferative activities.

Video S6. Representative application of C4-3D to visualize EdU signals (blue) in the kidney of 8-week-old mice, related to Figure 9 Green signals represent autofluorescence.

Video S7. Representative application of C4-3D to visualize EdU signals (blue) in the liver of 8-week-old mice, related to Figure 9 Green signals represent autofluorescence.

Video S8. Representative application of C4-3D to visualize EdU signals (blue) in the lung of 8-week-old mice, related to Figure 9 Green signals represent autofluorescence.

We also visualized EdU signals by C^4^-3D in mouse brains at ages 3 days, 2 months, and 12 months (Figure 7J). At age 3 days, proliferating cells were observed throughout the brain. In contrast, at age 2 months, proliferating cells were enriched in the SVZs and their migratory pathways to the olfactory bulbs. At age 12 months, proliferating cells were restricted to the frontal part of the SVZs and the olfactory bulbs.

## Discussion

We developed a technique (C^4^-3D) to integrate the *in situ* click reaction in tissue clearing to extensively identify the proliferating cells in whole mouse brain in 3D (Figure 2). C^4^-3D could be applied to the brain tissue cleared by CUBIC (Figure 1D and 1E), iDisco+ (Figures S5A and S5B), and PACT (Figure S5C and S5D), and was compatible with immunostaining (Figures 1F and 5A–5C), chemical nuclear staining (Figures 1E and 5I), and a fluorescent reporter mouse (Figures 6A and S6A–S6C). We also confirmed that C^4^-3D with EdU could be applied to disease models including cerebral infarction (Figures 7A, 7B, and S6D–S6G), GBM (Figures 7E, 7F, and S6H–S6K), and metastatic brain tumor (Figure 7G), as well as to the kidney, the liver, the lung, and an embryo in normal mouse (Figures 7H–7J). Identification and normalization of the EdU-positive nuclear coordinates for the Allen Brain Atlas enabled the identification of brain region-specific enrichment of EdU-positive nuclei (Figures 3F and 3G).

Tamura et al. recently reported a method that combined click chemistry with either hydrophilic (CUBIC) or organic solvent-based (BABB) tissue-clearing systems using high concentrations of reducing agents, a high-osmolarity buffer system, and multiple copper ligands, enabling *in situ* click reactions to penetrate deep into whole mouse brains and kidneys.^16^ In contrast, our approach intentionally avoided such auxiliary conditions and relied solely on the core components of the copper-catalyzed azide-alkyne cycloaddition reaction—sodium L-ascorbate, copper ions, and alkyne dyes—demonstrating robust compatibility with hydrophilic (CUBIC), hydrophobic (iDisco+), and hydrogel-based (PACT) tissue-clearing protocols. This distinction highlights a chemically simplified yet broadly adaptable strategy for whole-brain click labeling.

The only driving force of the *in situ* click reaction is passive diffusion of reagents, and the molecular weights of reagents and the reaction temperatures are critical factors.^37^^,^^38^^,^^39^ Small copper ions were supposed to permeate faster than the other reagents and to be easily oxidized in lack of reducing reagents. We were inspired by the SWITCH scheme, in which an antibody was uniformly permeated in the tissue at a low temperature, in which the low temperature suppressed the antibody-antigen binding.^40^ Then, the reaction temperature was increased to initiate the antibody-antigen binding. In C^4^-3D, we allowed fluorescent azide dye and SL-AA in the absence of copper ions to permeate in advance in the pre-incubation phase. Then, the Cu/SL-AA solution including a fluorescent azide dye, SL-AA, and copper ions were added so that copper ions permeated into the brain and initiated the *in situ* click reaction under a reduced environment. In addition, the Cu/SL-AA solution was refreshed in 48 h to prevent the formation of dehydroascorbic acid and abnormal pigmentation.

Counting the number of cells in the mammalian central nervous system has always been one of the most fundamental and technically challenging tasks in neuroanatomy.^41^^,^^42^ Two features are essential to accurately count the number of cells in a specific brain region. First, the signal-to-noise ratio should be high enough to appropriately recognize each nucleus. The accuracies to differentiate signals and noises in C^4^-3D were more than 0.988 (Table S1). Second, the spatial resolution should be high enough to distinctively recognize two adjacently located cells, especially newly divided cells.^43^^,^^44^ However, at the highest spatial resolution, the file becomes unreasonably huge in size and requires high computational resources and analysis time.^45^ We reduced the spatial resolution from 0.344 to 3.65 μm, while the accuracies of recognizing adjacently located nuclei were more than 0.878 (Table S2).

Automated normalization of the nuclear coordinates for the Allen Brain Atlas disclosed brain region-specific enrichment of EdU-positive nuclei (Figures 3F and 3G). To further delineate the brain region-specific enrichment of proliferating neuronal progenitors, we made SVZ-annotated and nestin-annotated versions of the Allen Brain Atlas (Data S1 and S2, respectively), which will serve as open-source platforms for analyzing the origins and the fates of proliferating neuronal progenitors.

Dormant neuronal progenitors become active and participate in brain repair processes after brain injury. We suppose that we were the first to demonstrate the spatial distribution of newly generated cells after brain infarction in 3D (Figures 7A, 7B, and S6D–S6G). Mapping of the nuclear coordinates to the Allen Brain Atlas showed regional enrichment of proliferative neuronal progenitors on five days after cerebral infarction (Figures 7C and 7D). Newly formed neuronal cells in the SVZ migrate at the speed of 17.98 ± 0.57 μm/h after cerebral infarction.^46^ In our mouse model of cerebral infarction, EdU was injected 24 h before being sacrificed and the migration distance of newly generated neurons was expected to be 431.52 μm in 24 h. Thus, migrating neuronal progenitors proliferated in the course of migration to the target region. Timed pulse-chase of EdU along with immunostaining for specific neuronal differentiation markers will be able to trace the proliferation, migration, and differentiation of neuronal progenitors after cerebral infarction. The other applications of C^4^-3D were mouse models of xenotransplanted GBM (Figures 7E, 7F, and S6H–S6K) and metastatic brain tumor (Figure 7G). Again, timed EdU pulses may delineate the progression and migration of the cancer cells in 3D. In addition, coimmunostaining may disclose the temporospatial associations with immune cells and blood vessels.

Although C^4^-3D can be applied to normal mice and mouse models of various diseases, some limitations must be noted. For example, certain disease models such as cerebral infarction may cause massive structural changes in the brain over an extended period, which makes automated normalization of nuclear coordinates difficult.^47^ Another example is that large organs such as the liver and the lungs need to be shrunk to fit in the constraint of LSFM. In addition, subsequent analyses of these organs require high computational resources. To summarize, C^4^-3D along with its downstream machine learning-based mapping of nuclear coordinates to specific brain regions has a potential to be applied to diverse fields of biomedical sciences. The compatibility of C^4^-3D with three major tissue-clearing methods of CUBIC, iDisco+, and PACT also has a potential to be applied to the analysis of hydrophilic and hydrophobic molecules.

### Limitations of the study

Although C^4^-3D enables robust whole-organ visualization of DNA synthesis, several limitations remain. First, we observed strong cortical signals in the brain without EdU injection. This was likely caused by accumulation of unbound Azide Dye647 in the brain surface and also by absorption of excitation light during LSFM imaging. This surface artifact complicates the interpretation of signal specificity, especially in peripheral brain regions. Second, while the method was compatible with various clearing techniques and tissues, performance may vary depending on tissue composition, fixation quality, or dye penetration efficiency. Third, we proposed that timed pulse-chase of EdU along with immunostaining for specific neuronal differentiation markers will be an attractive application of C^4^-3D to trace the proliferation, migration, and differentiation of neuronal progenitors, but have not shown any results of such experiments.

## Resource availability

### Lead contact

Requests for further information and resources should be directed to and will be fulfilled by the lead contact, Kinji Ohno (ohnok@med.nagoya-u.ac.jp).

### Materials availability

All unique/stable reagents generated in this study are available from the lead contact with a completed materials transfer agreement.

### Data and code availability

• All data reported in this paper will be shared by the lead contact upon request.
• MATLAB code for brain annotation and visualization is available at https://doi.org/10.6084/m9.figshare.29815871.
• Any additional information required to re-analyze the data reported in this paper is available from the lead contact upon request.

## Acknowledgments

We would like to thank Dr. Itaru Imayoshi at Graduate School of Biostudies, Kyoto University, and Dr. Ryoichiro Kageyama at RIKEN Center for Brain Science for providing us with Nestin-Cre/ERT2 transgenic mice; Dr. Fumiharu Ohka at Nagoya University Graduate School of Medicine for his technical advice; Dr. Issey Takahashi at Institute of Transformative Bio-Molecules, Nagoya University, for creating a graphical abstract; Ms. Eri Yorifuji and Ms. Mayumi Furukawa at Division for Medical Research Engineering, Nagoya University Graduate School of Medicine, for their technical assistance; and Mr. Akihiko Ichikawa at Carl Zeiss Co., Ltd., for his technical assistance. This work was supported by Grants-in-Aids from the 10.13039/100009619Japan Agency for Medical Research and Development (AMED) (JP23ek0109678), the 10.13039/501100001691Japan Society for the Promotion of Science (JSPS) (JP22K15394, JP23H02794, and JP23K18273), the 10.13039/501100003478Ministry of Health, Labour and Welfare of Japan (MHLW) (23FC1014), and the 10.13039/501100009438National Center of Neurology and Psychiatry (NCNP) (5–6). F.Z. was supported by a fellowship program by the THERS Interdisciplinary Frontier Next Generation Researcher.

## Author contributions

F.Z. and T.H. designed the study. F.Z. conducted most of the experiments and data analysis with the help of T.H., R.E., and A.E. T.H. generated a mouse model of GBM. F.Z. wrote the original manuscript, and T.H. and K.O. revised the manuscript. T.H. and K.O. provided funding support. T.H. and K.O. are responsible for the authenticity of the article and the sources of the data. All authors agreed to the content of the manuscript.

## Declaration of interests

The authors declare no competing interests.

## STAR★Methods

### Key resources table

**Table:** 

REAGENT or RESOURCE	SOURCE	IDENTIFIER
**Antibodies**		

anti-tyrosine hydroxylase	Millipore, Cat# AB152	RRID: AB_390204
goat anti-rabbit IgG (H + L) cross-absorbed secondary antibody, Alexa Fluor 546	Invitrogen, Cat# A11010	RRID: AB_2534077
Ki-67 monoclonal antibody (SolA15), eFluor 660, eBioscience	Invitrogen, Cat# 50-5698-82	RRID: AB_10854564
alpha-smooth muscle actin monoclonal antibody (1A4), eFluor 660, eBioscience	Invitrogen, Cat# 50-9760-82	RRID: AB_2574362

**Chemicals, peptides, and recombinant proteins**		

5-ethynyl-2′-deoxyuridine	Fujifilm, Cat# E915020	CAS RN: 15176-29-1
SYTOX Green Nucleic Acid Stain	Invitrogen, Cat# S7020	N/A
TO-PRO-3 Iodide (642/661)	Invitrogen, Cat# T3605	CAS RN: 157199-63-8
L-histidine	Tokyo Chemical Industry, Cat# H0149	CAS RN: 71-00-1
3-(4-((Bis((1-(*tert*-butyl)-1H-1,2,3-triazol-4-yl)methyl)amino)methyl)-1H-1,2,3-triazol-1-yl)propan-1-ol, BTTP	Click Chemistry Tools, Cat# 1414	CAS RN: 1341215-17-5
Tetrakis(acetonitrile)copper(I) Hexafluorophosphate	Tokyo Chemical Industry, Cat# T2665	CAS RN: 64443-05-6
AZDye 647 Azide Plus	Click Chemistry Tools, Cat# 1299-1	N/A
AZDye 568 Azide Plus	Click Chemistry Tools, Cat# 1480-1	N/A
Sodium L-Ascorbate	Tokyo Chemical Industry, Cat# A0539	CAS RN: 134-03-2
Copper(II) sulfate	Sigma-Aldrich, Cat# C1297	CAS RN: 7758-98-7
2NA(EDTA・2Na)	DojinDo, Cat# 6381-92-6	CAS RN: 6381-92-6
VA-044	Fujifilm, Cat# 225-02111	CAS RN: 27776-21-2
Tissue-Clearing Reagent CUBIC-L [for Animals]	Tokyo Chemical Industry, Cat# T3740	N/A
Tissue-Clearing Reagent CUBIC-R+(N) [for Animals]	Tokyo Chemical Industry, Cat# T3983	N/A
antipyrine	Tokyo Chemical Industry, Cat# D1876	CAS RN: 60-80-0
nicotinamide	Tokyo Chemical Industry, Cat# N0078	CAS RN: 98-92-0
N-butyldiethanolamine	Tokyo Chemical Industry, Cat# B0725	CAS RN: 102-79-4
Triton X-100	Sigma-Aldrich, Cat# T8787	CAS RN: 9036-19-5
Matrigel	CORNING, Cat#356234	CAS RN:119978-18-6
4% Paraformaldehyde	Fujifilm, Cat#161-20141	CAS RN:30525-89-4
casein	Fujifilm, Cat# 030-01505	CAS RN: 9000-71-9
sodium azide	Sigma-Aldrich, Cat# S2002	CAS RN:26628-22-8
Methanol	Fujifilm, Cat# 137-01823	CAS RN: 67-56-1
Dichloromethane	Sigma-Aldrich, Cat# 270997	CAS RN: 75-09-2
Dibenzyl ether	Sigma-Aldrich, Cat# 33630	CAS RN: 103-50-4
Ethyl (E)-Cinnamate	Tokyo Chemical Industry, Cat# C0359	CAS RN: 4192-77-2
Dimethyl Sulfoxide	Fujifilm, Cat# 049-07213	CAS RN: 67-68-5
Tamoxifen	LGC, Cat# 10540-29-1	CAS RN: 10540-29-1
sutures	RWD Life Science, Cat# MSMC23B100PK50	N/A
DMEM	Merck Millipore D6429	N/A
fetal bovine serum	Serena S-FBS-CO-015	N/A
penicillin-streptomycin	Fujifilm, Cat# 168-23191	CAS RN: 8025-06-7

**Critical commercial assays**		

Click-&-Go Plus 568 Imaging Kit	Vector Laboratories, Cat# CCT-1318	N/A
Click-&-Go Plus 647 Imaging Kit	Vector Laboratories, Cat# CCT-1320	N/A

**Experimental models: Cell lines**		

LN-229 (human glioblastoma)	ATCC, Cat# CRL-2611	RRID: CVCL_0393
LLC mouse cell line	RIKEN BRC Cat# RCB0558	RRID: CVCL_4358

**Experimental models: Organisms/strains**		

C57BL/6J mouse	CLEA Japan Inc	IMSR_JAX:000664
NOD/ShiJic-scid mouse	CLEA Japan Inc	https://www.clea-japan.com/en/products/immunodeficiency/item_a0040
Nestin-Cre/ERT2 mouse	Imayoshi et al.^32^	IMSR_JAX:016261
cRosa26-lsl-tdTomato mouse	Madisen et al.^33^	IMSR_JAX:007914

**Software and algorithms**		

ZEN (Blue Edition or Black Edition)	Zeiss	RRID: SCR_013672
Imaris 10.0	Oxford Instruments	RRID: SCR_007370
Fiji	Schindelin et al.^26^	RRID: SCR_002285
Labkit	Arzt et al.^25^	https://imagej.net/plugins/labkit/
3D Suite Segmentation tool	Ollion et al.^26^	https://imagej.net/plugins/3d-segmentation
antsRegistration	Klein et al.^48^	https://github.com/ANTsX/ANTs/wiki/Anatomy-of-an-antsRegistration-call
Allen Brain Atlas	Allen Institute for Brain Science	RRID: SCR_007416
MATLAB	MathWorks	RRID: SCR_001622
GraphPad Prism 10.1.2	GraphPad Software	RRID: SCR_002798
JSON Editor Online	Jos de Jong/jsoneditoronline.org	https://jsoneditoronline.org/
Brain annotation and visualization coding	this paper	https://doi.org/10.6084/m9.figshare.29815871

**Other**		

Model 940 Small Animal Stereotaxic Instrument with Digital Display Console	David Kopf Instruments Cat# Model 940	N/A

### Experimental model and study participant details

#### Animals

All mice studies were approved by the Animal Care and Use Committee of Nagoya University (approval #M240258-001). C57BL/6J and NOD/ShiJic-scid mice were purchased from CLEA Japan Inc. Nestin-Cre/ERT2 transgenic mice (RBRC05999, RIKEN) were kindly provided by Dr. Itaru Imayoshi at Kyoto University and Dr. Ryoichiro Kageyama at RIKEN Center for Brain Science.^32^^,^^33^ cRosa26-lsl-tdTomato mice (JAX stock #007909) were obtained from Jackson Laboratory.^33^ All mice were housed under a specific pathogen-free condition with a 12-h light/dark cycle and with *ad libitum* access to food and water. Environmental conditions were controlled with a temperature at 22 ± 2°C and a humidity of 55% ± 10%. Eight-week-old mice of both sexes were used in most experiments unless otherwise specified. Mice were randomly assigned to experimental groups, and efforts were made to ensure balanced representation of sexes to minimize sex-related bias. All animals used were drug/test-naive and had not undergone any previous procedures.

#### Cells

LN-229 human glioblastoma cells (CRL-2611; ATCC, Manassas, VA, USA) and LLC mouse lung carcinoma cells (RCB0558; RIKEN BioResource Center, Tsukuba, Japan) were cultured in Dulbecco’s Modified Eagle’s Medium supplemented with 10% fetal bovine serum (FBS) and 1% penicillin-streptomycin at 37°C in a humidified atmosphere containing 5% CO_2_. Cells were passaged every 2–3 days upon reaching 70–80% confluency and maintained for at least one week prior to downstream experiments.

### Method details

#### EdU administration and preparation of the brain

In accordance with previously reported protocols,^22^^,^^49^ EdU (E915020, Fujifilm) was prepared at 10 mg/mL in PBS at pH 7.4. EdU (100 μg/g body weight) was intraperitoneally administered three times in 24 h. At 24 h after administration of the last EdU, mice were sacrificed under deep anesthesia with isoflurane. For CUBIC and iDisco+, 4% paraformaldehyde (PFA) in PBS was administered for tissue fixation. Dissected brain was further fixed overnight at 4°C in 4% PFA in PBS and then for 1 h at room temperature. For PACT,^50^ mice was subjected to initial perfusion with cold 0.1 M PBS followed by a second perfusion with a cold hydrogel solution composed of 4% PFA, 4% acrylamide, 0.05% bis-acrylamide, and 0.25% VA-044 initiator (Fujifilm) in 0.1 M PBS. After perfusion, the brain was excised out and incubated in a fresh hydrogel solution at 4°C for three days. The hydrogel solution was then gassed with 100% nitrogen for 10 min and incubated at 37°C for 3 h to induce polymerization. After polymerization, the hydrogel was trimmed to extract the brain for further processing.

#### Tissue clearing with clear, unobstructed brain/body imaging cocktails and computational analysis (CUBIC)

The brain fixed in PFA was washed three times at room temperature in PBS to remove residual fixatives.^51^ The brain was then incubated in the CUBIC-L solution (T3740, Tokyo Chemical Industry) containing 10% N-butyldiethanolamine and 10% Triton X-100 for delipidation and decolorization on a rotary shaker at 37°C for five days with the solution being added every other day. After delipidation, the brain was briefly rinsed three times in PBS at room temperature. The brain was then stained by the *in situ* click reaction, which will be stated in detail below. For additional antibody staining, the brain was incubated with a primary or secondary antibody in a buffer containing 0.5% Triton X-100, 0.25% casein, and 0.01% sodium azide in PBS at 37°C for five days. The CUBIC-R solution (T3983, Tokyo Chemical Industry) contained 45% antipyrine and 30% nicotinamide. To match the refractive index, the brain was incubated in 50% CUBIC-R solution for one day followed by 100% CUBIC-R solution until the brain became completely transparent.

#### Tissue clearing with immunolabeling-enabled three-dimensional imaging of solvent-cleared organs (iDisco+)

The brain was first dehydrated with increasing concentrations of methanol from 20% to 100%.^47^ After dehydration, the brain was submerged in a 66% dichloromethane (DCM) and 33% methanol solution for 16 h to enhance lipid removal. The brain was then bleached overnight at 4°C in 5% hydrogen peroxide in methanol to remove pigments and autofluorescence. After bleaching, the brain underwent rehydration with decreasing concentrations of methanol from 80% to 0% in water, and were thoroughly washed with PBS to remove any residual solvents. The brain was then stained by the *in situ* click reaction, which will be stated below. An additional antibody staining protocol was the same as that of CUBIC. The brain was then immersed in dibenzyl ether or ethyl cinnamate (ECi)^52^ until they become completely transparent.

#### Tissue clearing with passive CLARITY technique (PACT)

The brain was incubated in 40 mL of a clearing solution containing 8% sodium dodecyl sulfate (SDS) in PBS at pH 7.5 on a rotatory shaker at 60°C.^50^ The clearing solution was replaced every 3–5 days, until the brain became clear in 12–14 days. The tissue-cleared brain was washed twice with PBS containing 0.1% Triton X-100 for ∼12 h each to remove any residual SDS and enhance the penetration of staining solutions. The *in situ* click reaction will be stated below. An additional antibody staining protocol was the same as that of CUBIC. After staining, the brain was immersed in a refractive index matching solution until they become completely transparent.

#### Measurement of oxidation-reduction potential (ORP)

ORP was measured using the LAQUA System (Horiba) following the manufacturer’s guidelines. The system was equipped with a platinum electrode (9300-10D, Horiba) that was specifically designed to measure ORP in solutions. The electrode was submerged at least 3 cm into the sample solution. Calibration was performed using standard solutions every day. The effects of ORP on the *in situ* click reaction were examined in cryosectioned tissues in 2D. As exposure to the air gradually increased the ORP of 0.1 M SL-AA solution, we left the SL-AA solution at room temperature to obtain the desired ORP levels. EdU signals in the tissue slices were visualized by the fist click reaction with the SL-AA solutions with AZDye 647 Azide Plus (1299-1, CCT) at different ORP levels. Subsequently, the slices were rigorously washed, and the second click reaction was conducted using Click-&-Go Plus 568 Imaging Kit (CCT-1318, Vector Laboratories). The intensity and uniformity of the signal of the first and second click reactions were analyzed under a confocal microscope.

#### Optimization of the *in situ* click reaction by inspecting the signal-to-noise ratio in 3D and by the second click reaction of cryostat sections in 2D

The optimization of the *in situ* click reaction was composed of two tracks (Figure S2). In the first track, the brain that was tissue cleared by CUBIC or iDisco+ was subjected to a conditional *in situ* click reaction with Azide Dye 568, and a 3D image was acquired with LSFM. Signal intensities on a line along the SVZ-brain surface or the olfactory bulb-SVZ-thalamus were analyzed with ZEN program (Zeiss). The conditions of the *in situ* click chemistry were optimized by inspecting the EdU signals and the background autofluorescence on the lines (Figure S2A). In the second track, after acquiring a 3D image with LSFM, the brain was fixed in 1% PFA, washed three times with PBS, and submerged in 30% sucrose in PBS. Depending on the fragility of the brain, 15 to 30 μm slices were obtained by a cryostat. Subsequently, the sliced tissue was subjected to the conventional click reaction with Click-&-Go Plus 647 Imaging Kit (CCT-1320, Vector Laboratories). By comparing the EdU signals in the 1st conditional *in situ* click reaction in 3D and in the 2nd conventional click reaction in 2D, we inspected whether the conditional *in situ* click reaction reached deep in the brain (Figure S2B).

#### Optimized *in situ* click reaction (C^4^-3D)

The tissue-cleared brain was preincubated in an SL-AA solution containing 0.1 M SL-AA (A0539, TCI), 0.2% Triton X-100, and 30 ng/mL (for whole adult brain) of AZDye 647 Azide Plus (1299-1, CCT) or AZDye 568 Azide Plus (1480-1, CCT) but without copper ions in PBS. The brain tissue cleared by CUBIC or PACT was preincubated at 25°C overnight, whereas the brain tissue cleared by iDisco+ was preincubated at 4°C for two days. The brain was then added with an SL-AA solution with 1 mM CuSO_4_, and was placed on a rotary shaker at 100 rpm at 37°C to initiate the *in situ* click reaction. After two days, a new SL-AA solution with 1 mM CuSO_4_ was added. The reaction was quenched by immersing the brain in 4 mM EDTA in PBS (pH 8.0) at 37°C for 6 h. The brain was then washed with PBS three times at room temperature to remove any unreacted reagents. Additional immunostaining was performed with anti-tyrosine hydroxylase (1:200, AB152, Millipore) and goat anti-rabbit IgG (H + L) cross-absorbed secondary antibody, Alexa Fluor 546 (1:200, A11010, Invitrogen) for CUBIC, iDisco+, and PACT; Ki-67 monoclonal antibody (SolA15), eFluor 660, eBioscience (1:200, 50-5698-82, Invitrogen) for CUBIC and iDisco+; and alpha-smooth muscle actin monoclonal antibody (1A4), eFluor 660, eBioscience (1:200, 50-9760-82, Invitrogen) for CUBIC and iDisco+. Chemical nuclear staining was performed with SYTOX Green Nucleic Acid Stain (1:500, S7020, Invitrogen) for CUBIC and TO-PRO-3 Iodide (642/661) (1:500, T3605, Invitrogen) for iDisco+.

We also evaluated the effects of copper ligands on the *in situ* click reaction. BTTP (1414, CCT) was used at a ratio of [CuSO_4_]:[BTTP] = 1:2. Similarly, L-histidine (H1049, TCI) was used at a ratio of [CuSO_4_]:[L-histidine] = 1:2. We also used monovalent copper, Tetrakis (acetonitrile)copper (I) Hexafluorophosphate (T2665, TCI), and tried to optimize its concentration from 1 to 10 mM for the *in situ* click reaction, but in vain.

#### Light sheet fluorescence microscopy (LSFM)

The C^4^-3D brain was first matched for its refractive index with their respective observation buffers. The brain was rotated on the mounting frame of the ZEISS Lightsheet 7 fluorescence microscope to align them perpendicular to the objective lens. The magnification was then adjusted according to the resolution requirements, followed by setting the field of view for a single capture. At this point, the ZEN program automatically divides the entire brain into rectangular blocks that can be stitched together. We set an overlap of 15% to ensure that no brain regions were lost in subsequent analysis. Next, the range of z-scanning was specified by defining the distance from the ventral to dorsal sides. Scanning proceeds from the dorsal to ventral sides of the brain with z stack intervals ranging from 0.245 to 3.65 μm, after which the magnification was adjusted to make the XY and Z resolutions identical. Fluorescence images were captured under 488 nm excitation for autofluorescence or nuclear staining, and under 594 or 647 nm excitation for EdU labeling or antibody staining.

The acquired images were processed using the ZEN program (Zeiss) for bilateral fusion, stitching, and optional deconvolution. The acquired images were visualized with Imaris 10.0 software (Oxford Instruments).

#### Machine learning-based distinction of signals and noises and identification of each nucleus

With the Labkit plugin^25^ for Fiji,^26^ hundreds of clusters of EdU signals in at least ten brain slices were manually annotated as nuclei (“foreground”) or noises (“background”) for each mouse brain (Step ii in Figure 3B). A random forest modeling tool in Labkit made a model to differentiate nuclei and noises. The accuracy of the model was validated by comparing the model-generated signal/noise labels and the manually curated signal/noise labels in about ten brain slices in five brain regions that were not used to generate a model.

We next used the 3D Suite Segmentation tool^27^ for Fiji^26^ to identify each nucleus even when two or more nuclei were closely adjacent each other (Step iv in Figure 3B). The accuracy of segmentation was validated by comparing the predicted numbers of nuclei and the manually counted numbers of nuclei in about ten brain slices in five brain regions that were not used to generate a model.

#### Normalization of the nuclear coordinates for those in the Allen Brain Atlas

We first obtained the average template brain data and its annotation data from the Allen Brain Atlas as a reference. We set the coordinate of the most left-anterior-superior voxel of the reference atlas to (0, 0, 0). Subsequently, we matched the voxel sizes of the autofluorescence images to those of the reference atlas with Fiji.^26^ We then employed the image registration algorithm (rigid, affine, and SyN transformations) of the antsRegistration on Linux (https://github.com/ANTsX/ANTs/wiki/Anatomy-of-an-antsRegistration-call)^53^ to align the autofluorescence images to the reference atlas. We visually verified the aligned image (M2F_1Warp.nii.gz) and obtained the forward affine transformation matrix (M2F_0GenericAffine.mat) and the inverse nonlinear deformation field (M2F_1InverseWarp.nii.gz) for the transformation of nuclear coordinates. Specifically, the affine matrix (M2F_0GenericAffine.mat) was used inversely to map the coordinates of the sample space to those of the reference atlas, while the inverse deformation field (M2F_1InverseWarp.nii.gz) was directly applied for the same purpose.

#### Construction of proliferation atlas of the mouse brain at a single nucleus resolution

The normalized nuclear coordinates have the corresponding voxel coordinates in the Allen Brain Atlas, and each voxel coordinate has its brain region ID. The 71 major brain regions at the top level of the brain structure hierarchy were used in our analysis. The number of EdU-positive nuclei was counted for each brain region, and was divided by the volume of the brain region to calculate the ratio of EdU-positive nuclei. An interactive 3D heatmap showing the ratios of EdU-positive nuclei was generated by Viewer3D in MATLAB.

#### Custom annotations of the Allen Brain Atlas

We introduced three custom annotations to the Allen Brain Atlas as open-source platforms.

The first customization was the SVZ-annotated brain atlas. By visually inspecting the previously reported definition of the mouse SVZ,^48^ we manually segmented a layer of single pixels on the lateral wall of the caudate nucleus region in 2D slices of the anatomical atlas (average template.nrrd) provided by the Allen Brain Atlas. The coordinates of the segmented voxels were determined by Fiji, and were marked with ID = 5 to represent the SVZ using the JSON editor at https://jsoneditoronline.org/. The SVZ-annotated brain atlas is provided in Data S1.

The second customization was the nestin-annotated brain atlas. Tamoxifen (10540-29-1, LGC) was prepared at 20 mg/mL in corn oil. Nestin-Cre/ERT2-Rosa26-lsl-tdTomato mouse was intraperitoneally injected with 200 mg/kg of tamoxifen for five consecutive days. The brain was tissue cleared by CUBIC and was subjected to C^4^-3D (Figure 4). The coordinates of nestin and EdU signals were normalized to those of the Allen Brain Atlas. Voxels that were positive for nestin in four or more times in seven mouse brains were given ID = 5 to represent nestin-positive voxels using the JSON editor at https://jsoneditoronline.org/. The nestin-annotated brain atlas is provided in Data S2.

The third customization was the hemisphere-annotated brain atlas. The Allen Brain Atlas has symmetrical brain IDs and no hemispheric information is included. We added 1,000,000,000 to the original brain IDs on the left hemisphere, while the brain IDs on the right hemisphere remained unedited. In the index, we added “right” or “left” on the name of the brain region, like “right dentate gyrus”.

#### Colocalization analysis of EdU and Ki-67 signals

The distance between the nuclear coordinates of EdU and Ki-67 signals was measured by MATLAB. Distances less than 5 μm were defined to be colocalized. A deformation matrix that was obtained by antsRegistration was applied to the nuclear coordinates to annotate the brain region for each nuclear coordinate.

#### Application of C^4^-3D to a mouse model of cerebral infarction

The left cerebral infarction was induced in C57BL/6J mice at age 8 weeks by occlusion of the left middle cerebral artery under anesthesia with isoflurane (4% for induction and 1.5–2.0% for maintenance). Under sterile conditions, a small incision was made in the neck to expose the left common carotid artery. A silicone-coated nylon filament (MSMC23B100PK50, RWD) was inserted through the external carotid artery and gently advanced into the internal carotid artery until it blocked the origin of the middle cerebral artery.^54^

#### Application of C^4^-3D to a mouse model of xenograft glioblastoma multiforme

Human LN-229 glioblastoma cells were obtained from ATCC. The cells were cultured in high-glucose Dulbecco’s Modified Eagle Medium (DMEM) (Gibco) supplemented with 10% FBS (Gibco) and 1% penicillin-streptomycin (15140122, Gibco). At 80–90% confluency, the cells were harvested, washed, trypsinized, and resuspended in PBS to a concentration of 5 × 10^5^ cells/μL. The cells (1 μL) were added with 1 μL Matrigel (Corning), and were implanted into the brain of an immunodeficient NOD/ShiJic-scid mouse (CLEA Japan). Under anesthesia with a combination of medetomidine (0.3 mg/kg), midazolam (4 mg/kg), and butorphanol (5 mg/kg),^55^ a small burr hole was drilled into the mouse skull, and the cell suspension (2 μL) was slowly injected into the striatum at the coordinates of AP 0.940 mm, ML -1.798 mm, and DV 3.000 mm using a fine, calibrated pipette attached to a stereotactic apparatus (Model 940, David Kopf Instruments). The needle was inserted up to DV 4.000 mm at first, and pulled back to DV 3.000 mm to form a cavity. After injection, the burr hole was sealed with bone wax, and the incision was sutured. After four weeks, the mouse brain was excised out under deep anesthesia with isoflurane.

#### Application of C^4^-3D to a mouse model of intracranial metastasis of lung cancer

LLC mouse cells (RIKEN BioResource Center) were cultured in high-glucose DMEM (Gibco) supplemented with 10% FBS and 1% penicillin-streptomycin. The cell suspension was carefully injected into the tail vein of C57BL/6J mouse. Two weeks after injection, metastatic brain tumor was analyzed.

#### Computational system

The computer system was equipped with Intel Core i7-13700K, NVIDIA RTX A5000 GPU, 192 GB DRAM, and 4 TB SSDs, and 24 TB HDDs, which enabled calculations of each 3D dataset in ∼3 (single signal channel) to ∼7 h (dual signal channels).

### Quantification and statistical analysis

#### Statistical analysis

All values are presented as mean ± standard deviation. Statistical significance was calculated by one-way ANOVA followed by Tukey’s post hoc test or paired *t*-test using Prism 10.3.1. (GraphPad Software). *p*-value of 0.05 or less was considered to be statistically significant.

## Supplemental information

Document S1. Figures S1–S6, Tables S1 and S2

Data S1. This dataset provides a manually annotated version of the Allen Brain Atlas, focusing on the SVZ in the caudoputamen subregion of the striatum, related to Figure 6B The SVZ is not included in the original Allen Brain Atlas, likely due to its thin structure consisting of only a few cell layers adjacent to the lateral ventricles. To address this omission, we manually segmented the SVZ by referencing previously reported definitions of the mouse SVZ. Using 2D slices of the anatomical atlas (average template.nrrd), a single layer of pixels was annotated on the lateral wall of the caudate nucleus region. The annotated voxels were assigned ID = 5 and incorporated into the atlas using a JSON editor.

Data S2. This dataset provides a customized version of the Allen Brain Atlas, focusing on nestin-positive signals to delineate neuronal progenitor migration pathways, related to Figure 6C Using Nestin-Cre/ERT2-Rosa26-lsl-tdTomato mice treated with tamoxifen (200 mg/kg intraperitoneally for five consecutive days), nestin expression was visualized following tissue clearing with CUBIC and the application of C4-3D. Coordinates of nestin-positive voxels were normalized to the Allen Brain Atlas. Voxels identified as nestin-positive in at least four out of seven mouse brains were annotated with ID = 5. This nestin-annotated atlas highlights the gradual decrease in nestin expression along migration pathways to the olfactory bulbs, enabling detailed spatial analysis.

Document S2. Article plus supplemental information
