# Supplementary material for: An optimized click chemistry method allows visualization of proliferating neuronal progenitors in the mouse brain
Source: Cell Rep Methods. 2025 Oct 21;5(11):101208. doi: 10.1016/j.crmeth.2025.101208 (PMC12664898; doi:10.1016/j.crmeth.2025.101208)
Supplement: Document S1. Figures S1–S6, Tables S1 and S2 [file mmc1.pdf]

Cell Reports Methods, Volume *5*

Supplemental information

An optimized click chemistry method allows visualization of proliferating neuronal progenitors in the mouse brain

Fei Zhao, Tomonari Hamaguchi, Ryo Egawa, Atsushi Enomoto, and Kinji Ohno

Supplemental Information

Tissue clearing coupled with click chemistry in 3D (C^4-3D) to visualize proliferating neuronal progenitors in whole mouse brain

Fei Zhao, Tomonari Hamaguchi, Ryo Egawa, Atsushi Enomoto, Kinji Ohno

Table S1. Differentiation of signals and noises by Labkit for Fiji, related to Figure 3

	Observed signals	Observed noises	Accuracy
**Cortex**			
Predicted signals	971	72	0.99996
Predicted noises	23	2,519,274	
**SVZ**			
Predicted signals	63,580	2867	0.99765
Predicted noises	1262	1,691,414	
**DG**			
Predicted signals	31,821	422	0.99969
Predicted noises	356	2,466,661	
**Glioblastoma**			
Predicted signals	1,124,931	14,982	0.99183
Predicted noises	2,494	997,362	
**Cerebral infarction**			
Predicted signals	1,445,213	22,114	0.98811
Predicted noises	3,012	643,145	

The observed and predicted pixels of signals and noises of ~10 brain slices of a single mouse brain are indicated.

Table S2. Identification of the centers of nuclei in a cluster of EdU signals, related to Figure 3

Observed # of nuclei	3	2	1	Accuracy
**Cortex**				
Predicated # of nuclei = 3	2	3	0	0.91525
Predicated # of nuclei = 2	0	19	2	
Predicated # of nuclei = 1	0	5	87	
**SVZ**				
Predicated # of nuclei = 3	12	62	0	0.87755
Predicated # of nuclei = 2	2	211	21	
Predicated # of nuclei = 1	0	5	422	
**DG**				
Predicated # of nuclei = 3	5	22	0	0.90228
Predicated # of nuclei = 2	0	66	7	
Predicated # of nuclei = 1	0	1	206	
**Glioblastoma**				
Predicated # of nuclei = 3	22	214	0	0.91928
Predicated # of nuclei = 2	27	3628	155	
Predicated # of nuclei = 1	0	132	2164	
**Cerebral infarction**				
Predicated # of nuclei = 3	24	112	0	0.92671
Predicated # of nuclei = 2	14	2150	241	
Predicated # of nuclei = 1	0	64	3055	

The observed and predicted numbers of adjacently located nuclei of approximately 10 brain slices are indicated for each brain region.

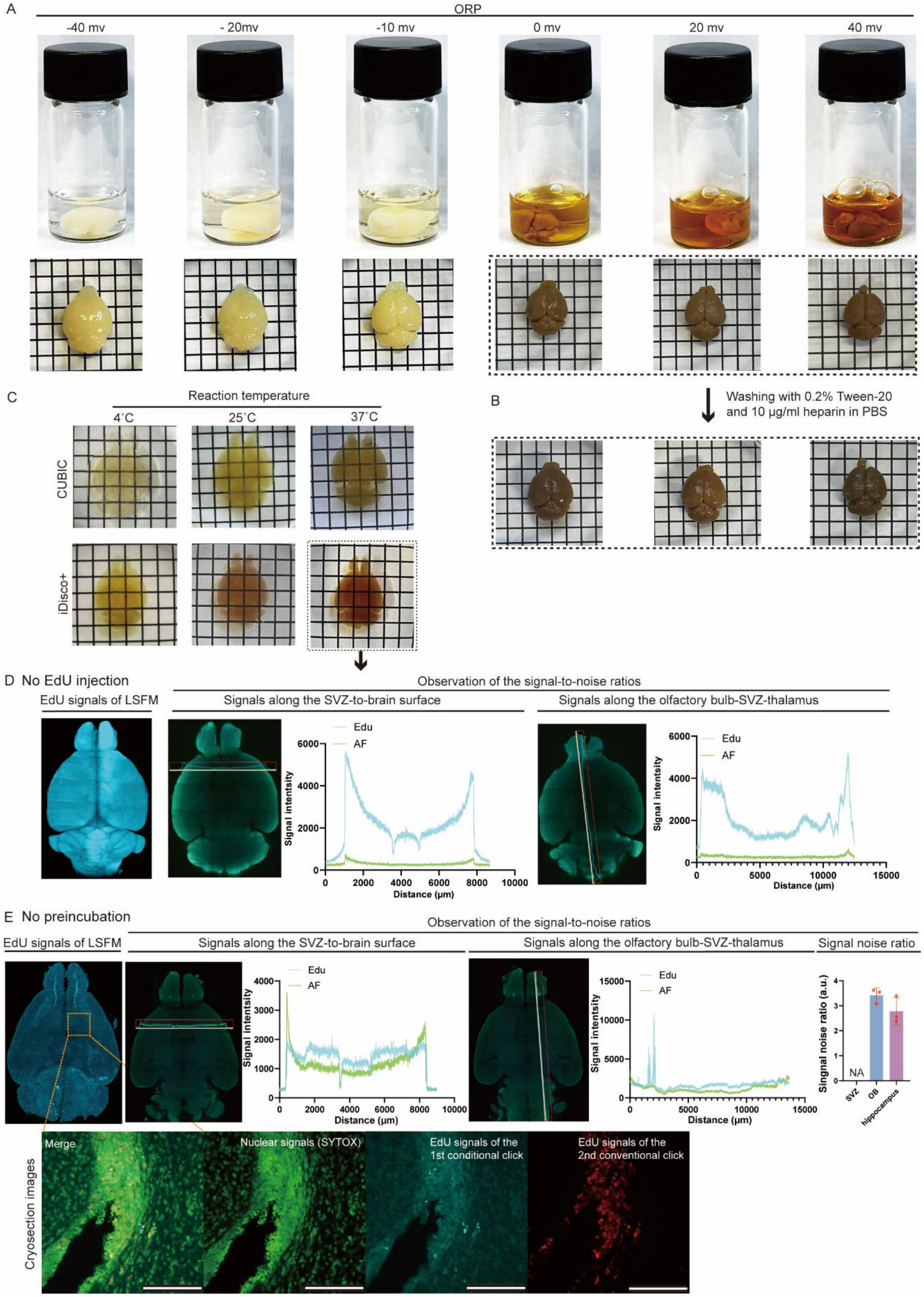
A
ORP
-40 mv
- 20mv
-10 mv
0 mv
20 mv
40 mv
C
Reaction temperature
4°C
25°C
37°C
CUBIC
iDisco+
B
Washing with 0.2% Tween-20 and 10 μg/ml heparin in PBS
D No EdU injection
Observation of the signal-to-noise ratios
EdU signals of LSFM
Signals along the SVZ-to-brain surface
Signals along the olfactory bulb-SVZ-thalamus
Edu
AF
Signal intensity
Distance (μm)
E No preincubation
Observation of the signal-to-noise ratios
EdU signals of LSFM
Signals along the SVZ-to-brain surface
Signals along the olfactory bulb-SVZ-thalamus
Signal noise ratio
Signal noise ratio (a.u.)
NA
SVZ
OB
hippocampus
Cryosection images
Merge
Nuclear signals (SYTOX)
EdU signals of the 1st conditional click
EdU signals of the 2nd conventional click

Figure S1. Oxidized forms of ascorbic acid cause abnormal pigmentation that cannot be cleared by additional tissue-clearing, related to Figure 1.

A Incubation of the tissue-cleared brain with sodium L-ascorbic acid (SL-AA) without copper ions caused pigmentation at increased ORP levels.

B Washing the pigmented brain samples with phosphate-buffered saline containing 0.2% Tween-20 and 10 μg/ml heparin (PTwH) for one day failed to remove the pigments. The pigments are a major source of high intrinsic fluorescence in transparent tissue samples.

C Incubation at increasing temperatures caused more pigmentation for both CUBIC and iDisco+.

D Negative control without EdU injection. (Left) Representative maximum intensity projection (MIP) image stacking the Z-axis of light-sheet fluorescent microscope (LSFM), showing autofluorescence signals detected by click chemistry in the absence of EdU injection. (Middle) Signal intensity profile along the subventricular zone (SVZ) to brain surface. (Right) Signal intensity profile along the olfactory bulb–SVZ–thalamus axis. Non-specific EdU signals, as well as autofluorescent (AF) signals, are higher in the cortical areas compared to central areas, because of absorption of the exciting light of LSFM on the brain surface. See **Figure S2A** for schematic diagram of the experiments. Similar cortical signals were observed in brains without EdU injection in repeated experiments, suggesting that unbound Azide Dye647 might have nonspecifically accumulated in surface regions, where stronger light absorption may further enhance the cortical signal appearance.

E *In situ* click reaction at 37 °C without SL-AA preincubation. Top panels show EdU signals (blue) and autofluorescence (AF, green) in 3D, as well as the signal intensity profiles along the SVZ-to-brain surface and olfactory bulb–SVZ–thalamus axes. Lower panels show a cryosectioned brain slice stained by the first (blue) and second (red) click reactions, and nuclei by SYTOX (green). Incomplete labeling and increased background were observed in the lack of preincubation. Scale bar = 100 μm. Signal-to-noise ratios (SNRs) were calculated for the SVZ, olfactory bulb (OB), and hippocampus and are shown in the rightmost bar graph. SNR was defined as the mean signal intensity divided by the mean background intensity. In cases where no positive signal was detected, SNR was not calculated and marked as “NA,” indicating that the value is not applicable. Each experimental group included three independent brain samples ($n = 3$ mice). Preincubation with SL-AA markedly improved the SNR of SVZ (see **Figure 1DE**). See **Figure S2B** for schematic diagram.

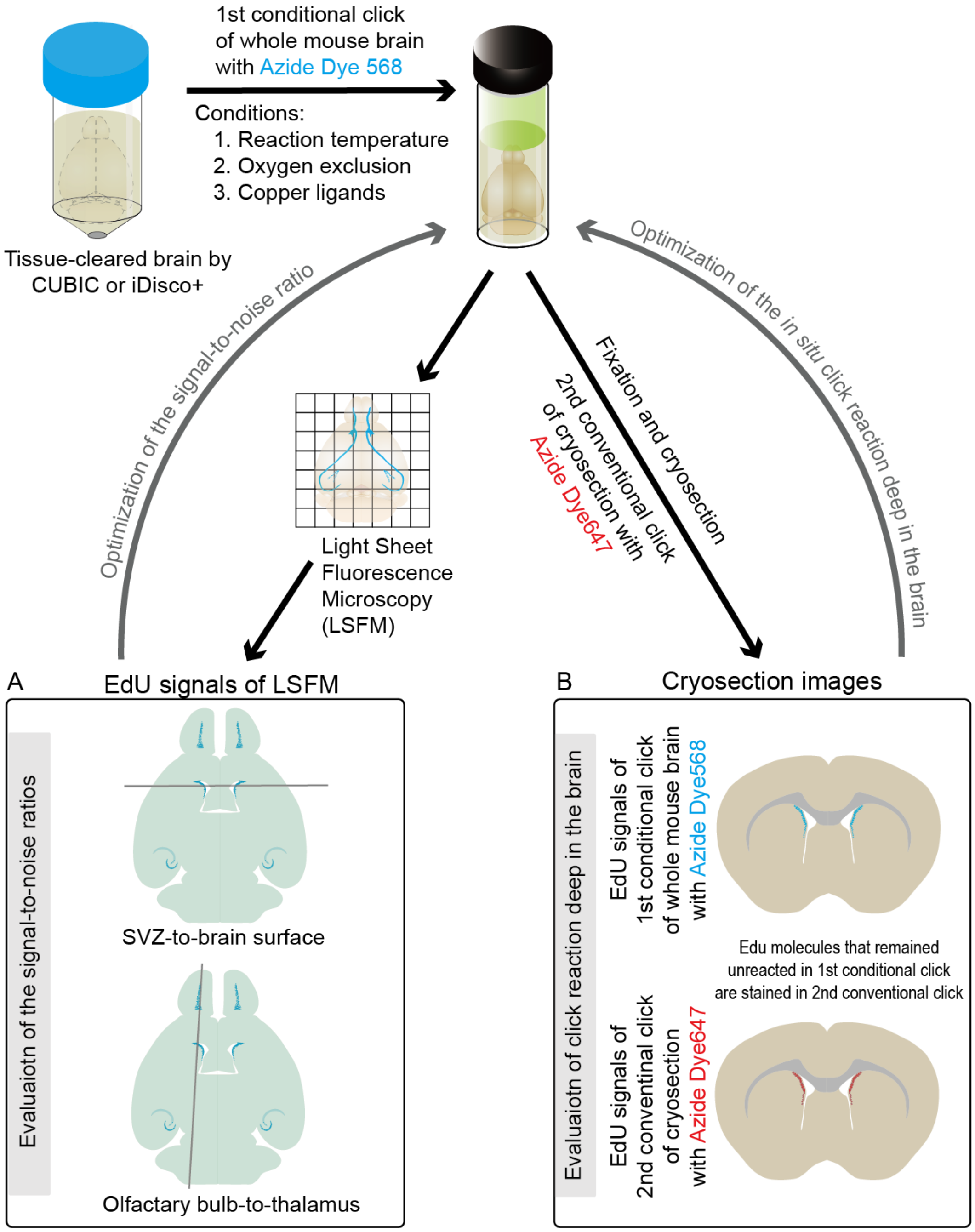

Figure S2. Schematic to optimize the conditions of in situ click reactions, related to Figure 1.

A The conditions of the first conditional click reaction were optimized to increase the signal-to-noise ratios on lines drawn on the SVZ-to-brain surface and the olfactory bulb-SVZ-thalamus of the captured 3D images. High autofluorescence on the surfaces of the olfactory bulbs was also used as background noises to be reduced. Note that although the first click reaction used Azide dye 568, which was excited under red fluorescence, we used blue to represent the 3D signal of Edu in tissue clearing for consistency in color matching throughout the paper.

B After the first conditional *in situ* click reaction, the brain was refixed, dehydrated, and cryosectioned. The second conventional click reaction was performed on the cryostat sections with Azide Dye 647 (red) to detect EdU that remained unreacted in the first click reaction. The conditions of the first click reaction were optimized to increase the blue signals deep inside the brain.

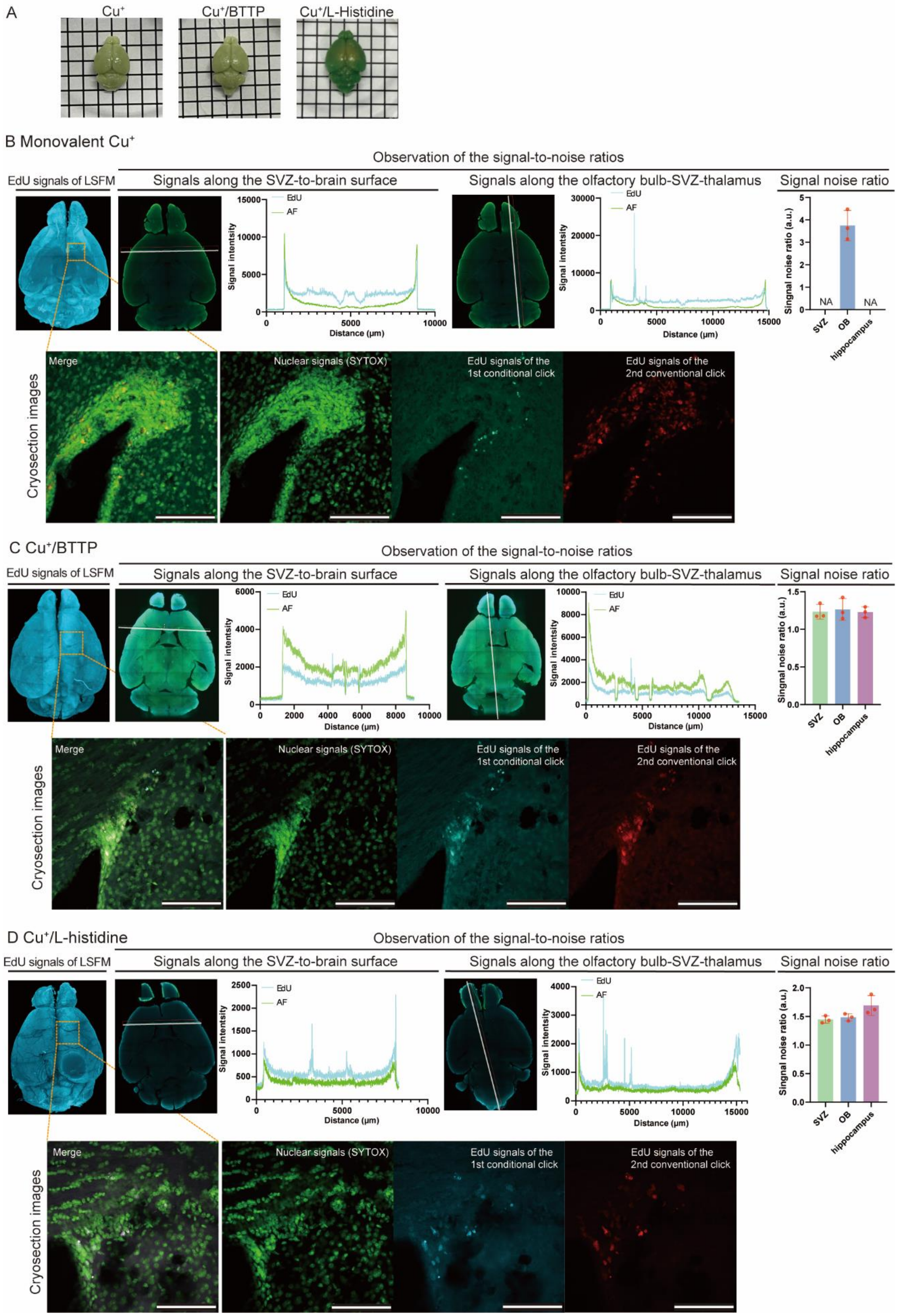
A
Cu⁺
Cu⁺/BTTP
Cu⁺/L-Histidine
B Monovalent Cu⁺
Observation of the signal-to-noise ratios
EdU signals of LSFM
Signals along the SVZ-to-brain surface
Signals along the olfactory bulb-SVZ-thalamus
Signal noise ratio
EdU
AF
Signal intensity
Distance (μm)
Singnal noise ratio (a.u.)
NA
SVZ
OB
hippocampus
Cryosection images
Merge
Nuclear signals (SYTOX)
EdU signals of the 1st conditional click
EdU signals of the 2nd conventional click
C Cu⁺/BTTP
Observation of the signal-to-noise ratios
EdU signals of LSFM
Signals along the SVZ-to-brain surface
Signals along the olfactory bulb-SVZ-thalamus
Signal noise ratio
Cryosection images
Merge
Nuclear signals (SYTOX)
EdU signals of the 1st conditional click
EdU signals of the 2nd conventional click
D Cu⁺/L-histidine
Observation of the signal-to-noise ratios
EdU signals of LSFM
Signals along the SVZ-to-brain surface
Signals along the olfactory bulb-SVZ-thalamus
Signal noise ratio
Cryosection images
Merge
Nuclear signals (SYTOX)
EdU signals of the 1st conditional click
EdU signals of the 2nd conventional click

Figure S3. Copper ligands (BTTP and L-histidine) do not improve the click reaction, related to Figure 1.

A Representative whole brain images stained by the indicated monovalent copper products.

B, C, D EdU in tissue-cleared brain sample was stained by monovalent Cu^+ **(B)**, Cu^+ coupled with BTTP ligands **(C)**, and Cu^+ coupled with L-histidine **(D)**. Top panels show representative maximum intensity projection (MIP) image by stacking the Z-axis of EdU signals (blue) and autofluorescence (AF) signals (green) taken by LSFM after the 1st conditional click reaction (see **Figure S2A** for schematic diagram). Lower panels show cryosections of the LSFM brain samples shown in top panels. The cryosections were stained for EdU by the second (red) conventional click reaction, as well as for the nuclei by SYTOX (green) (see **Figure S2B** for schematic diagram). Note that BTTP and L-histidine rather increased autofluorescence. Scale bar = 100 μm. Signal-to-noise ratios (SNRs) were calculated for the SVZ, olfactory bulb (OB), and hippocampus, and are shown in the rightmost bar graph. SNR was defined as the mean signal intensity divided by the mean background intensity. In cases where no positive signal was detected, SNR was not calculated and marked as "NA," indicating that the value is not applicable. Each experimental group included three independent brain samples (*n* = 3 mice).

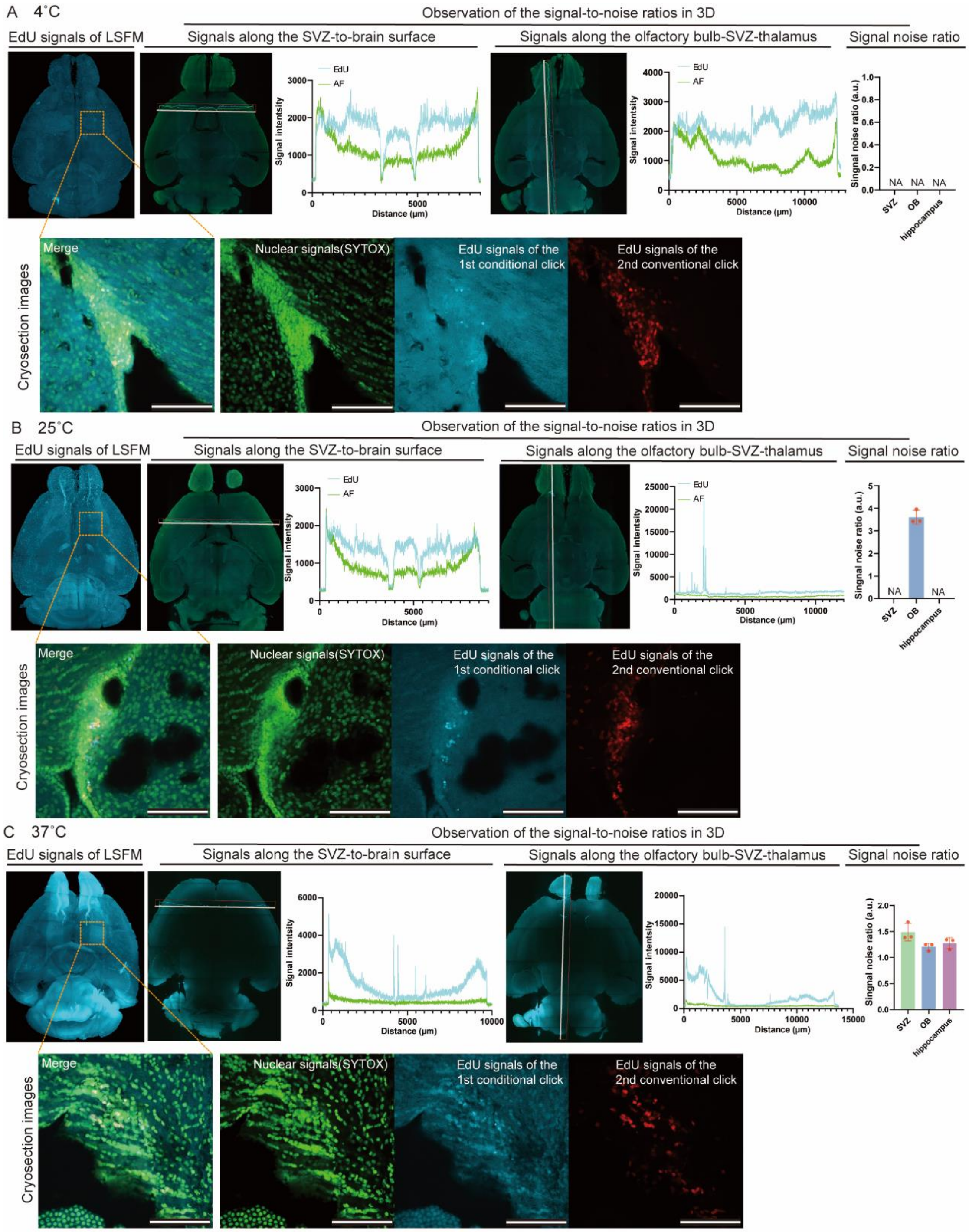

A 4˚C
Observation of the signal-to-noise ratios in 3D
EdU signals of LSFM
Signals along the SVZ-to-brain surface
Signals along the olfactory bulb-SVZ-thalamus
Signal noise ratio
EdU
AF
Signal intensity
Distance (μm)
Signal noise ratio (a.u.)
NA NA NA
SVZ
OB
hippocampus
Cryosection images
Merge
Nuclear signals(SYTOX)
EdU signals of the 1st conditional click
EdU signals of the 2nd conventional click
B 25˚C
Observation of the signal-to-noise ratios in 3D
EdU signals of LSFM
Signals along the SVZ-to-brain surface
Signals along the olfactory bulb-SVZ-thalamus
Signal noise ratio
Singnal noise ratio (a.u.)
C 37˚C
Observation of the signal-to-noise ratios in 3D
EdU signals of LSFM
Signals along the SVZ-to-brain surface
Signals along the olfactory bulb-SVZ-thalamus
Signal noise ratio

Figure S4. Click reaction at 37˚C gives rise to a high signal-to-noise ratio, related to Figure 1.

In situ click reaction was performed at 4˚C **(A)**, 25˚C **(B)**, and 37˚C **(C)**. In contrast to **Figure 1DE** that followed the optimized *in situ* click protocol of changing the staining solution at 48 h (**Figure 1C)**, the brain sample was soaked in the staining solution for 96 h without changing it at 48 h. Top panels show representative maximum intensity projection (MIP) image by stacking the Z-axis of EdU signals (blue) and autofluorescence (AF) signals (green) taken by LSFM after the 1st conditional click reaction (see **Figure S2A** for schematic diagram). Lower panels show cryosections of the LSFM brain samples shown in top panels. The cryosections were stained for EdU by the second (red) conventional click reaction, as well as for the nuclei by SYTOX (green) (see **Figure S2B** for schematic diagram). Note that Y scales showing signal intensities are different from graph to graph because of different maximum EdU signal intensities, which make AF signals ostensively look low or high. Signal-to-noise ratios (SNRs) were calculated for the SVZ, olfactory bulb (OB), and hippocampus, and are shown in the rightmost bar graph. SNR was defined as the mean signal intensity divided by the mean background intensity. Each experimental group included three independent brain samples (*n* = 3 mice). Note that the reaction at 37˚C gave rise to high EdU signals with low AF noises. Scale bar = 100 μm.

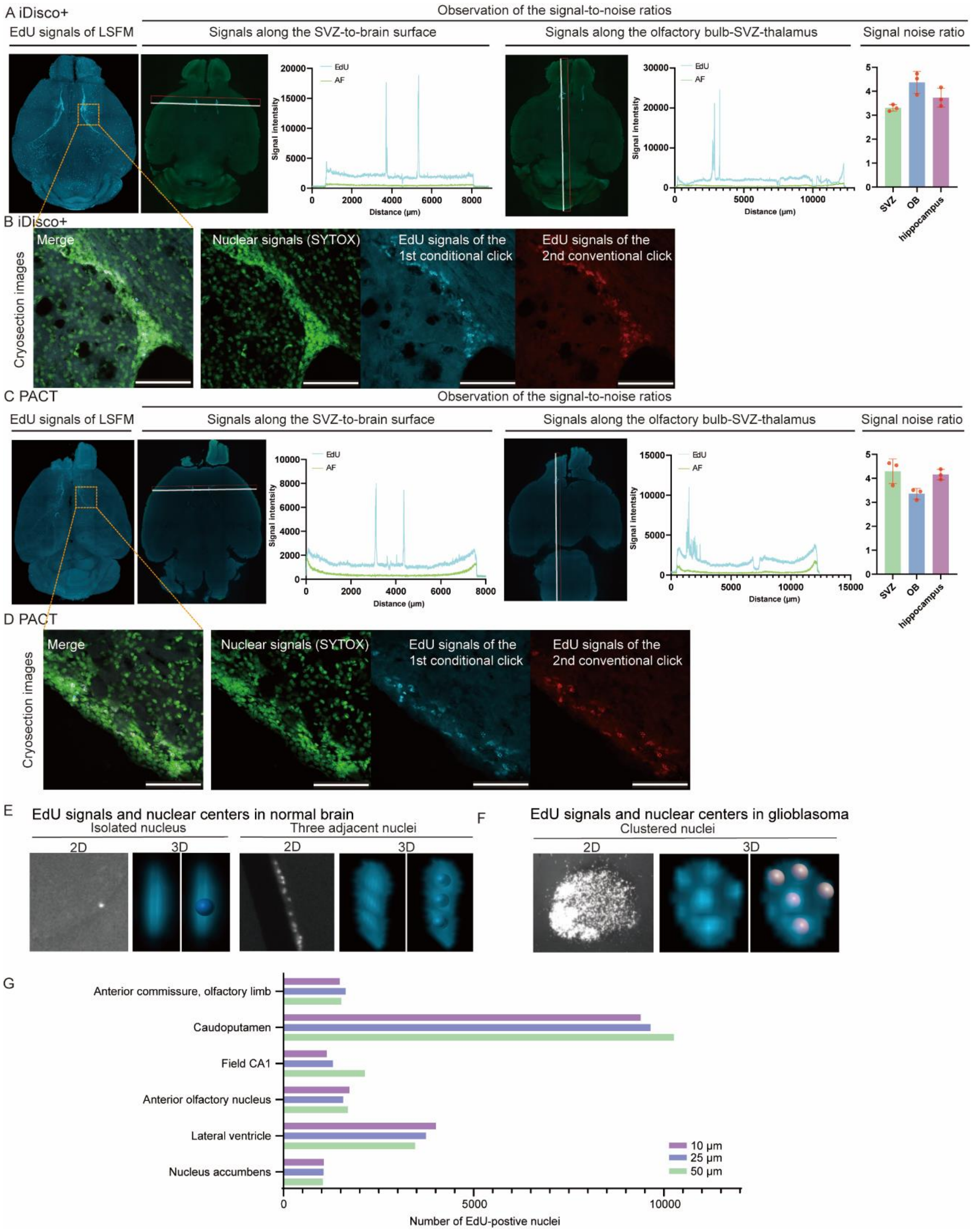
A iDisco+
Observation of the signal-to-noise ratios
EdU signals of LSFM
Signals along the SVZ-to-brain surface
Signals along the olfactory bulb-SVZ-thalamus
Signal noise ratio
EdU
AF
Signal intensity
Distance (μm)
SVZ
OB
hippocampus
B iDisco+
Cryosection images
Merge
Nuclear signals (SYTOX)
EdU signals of the 1st conditional click
EdU signals of the 2nd conventional click
C PACT
Observation of the signal-to-noise ratios
EdU signals of LSFM
Signals along the SVZ-to-brain surface
Signals along the olfactory bulb-SVZ-thalamus
Signal noise ratio
D PACT
Cryosection images
Merge
Nuclear signals (SYTOX)
EdU signals of the 1st conditional click
EdU signals of the 2nd conventional click
E
EdU signals and nuclear centers in normal brain
Isolated nucleus
Three adjacent nuclei
2D
3D
F
EdU signals and nuclear centers in glioblasoma
Clustered nuclei
G
Anterior commissure, olfactory limb
Caudoputamen
Field CA1
Anterior olfactory nucleus
Lateral ventricle
Nucleus accumbens
10 μm
25 μm
50 μm
Number of EdU-postive nuclei

Figure S5. Representative C⁴-3D images of whole brains tissue-cleared by iDisco+ (A, B) and PACT (C, D), related to Figure 1 and requirement of a voxel size of 10 μm in the Allen Brain Atlas to distinctively recognize each nucleus (E–G, related to Figure 3)

A, C (Left panels) Representative maximum intensity projection (MIP) image by stacking the Z-axis of EdU signals (blue) and autofluorescence (AF) signals (green) taken by LSFM after the 1st conditional click reaction (see **Figure S2A** for schematic diagram). Signal intensities on the indicated lines along the SVZ-to-brain surface (middle panels) and the olfactory bulb-SVZ-thalamus (right panels) are indicated. AF, autofluorescence. Signal-to-noise ratios (SNRs) were calculated for the SVZ, olfactory bulb (OB), and hippocampus and are shown in the rightmost bar graph. SNR was defined as the mean signal intensity divided by the mean background intensity. Each experimental group included three independent brain samples (*n* = 3 mice).

B, D EdU-stained brains in **A** and **C** were cryosectioned at the level of the orange dotted rectangle. The brain slices were stained by SYTOX (green) and by an additional conventional click reaction (see **Figure S2B** for schematic diagram). Note that equivalent images with CUBIC are indicated in **Figure 1DE**. Scale bar = 100 μm. Signal-to-noise ratios (SNRs) were calculated for the SVZ, olfactory bulb (OB), and hippocampus and are shown in the rightmost bar graph. SNR was defined as the mean signal intensity divided by the mean background intensity. Each experimental group included three independent brain samples (*n* = 3 mice).

E, F Representative EdU signals in 2D and 3D with or without a ball indicating the nuclear center in normal mouse brain **(E)** and glioblastoma **(F)**. The nuclear center was recognized by 3D Suite Segmentation tool[1].

G The numbers of EdU-positive nuclei at different voxel sizes at different brain regions in a single mouse brain. Note that EdU-positive nuclei in the lateral ventricle were erroneously classified into the caudoputamen at high voxel sizes.

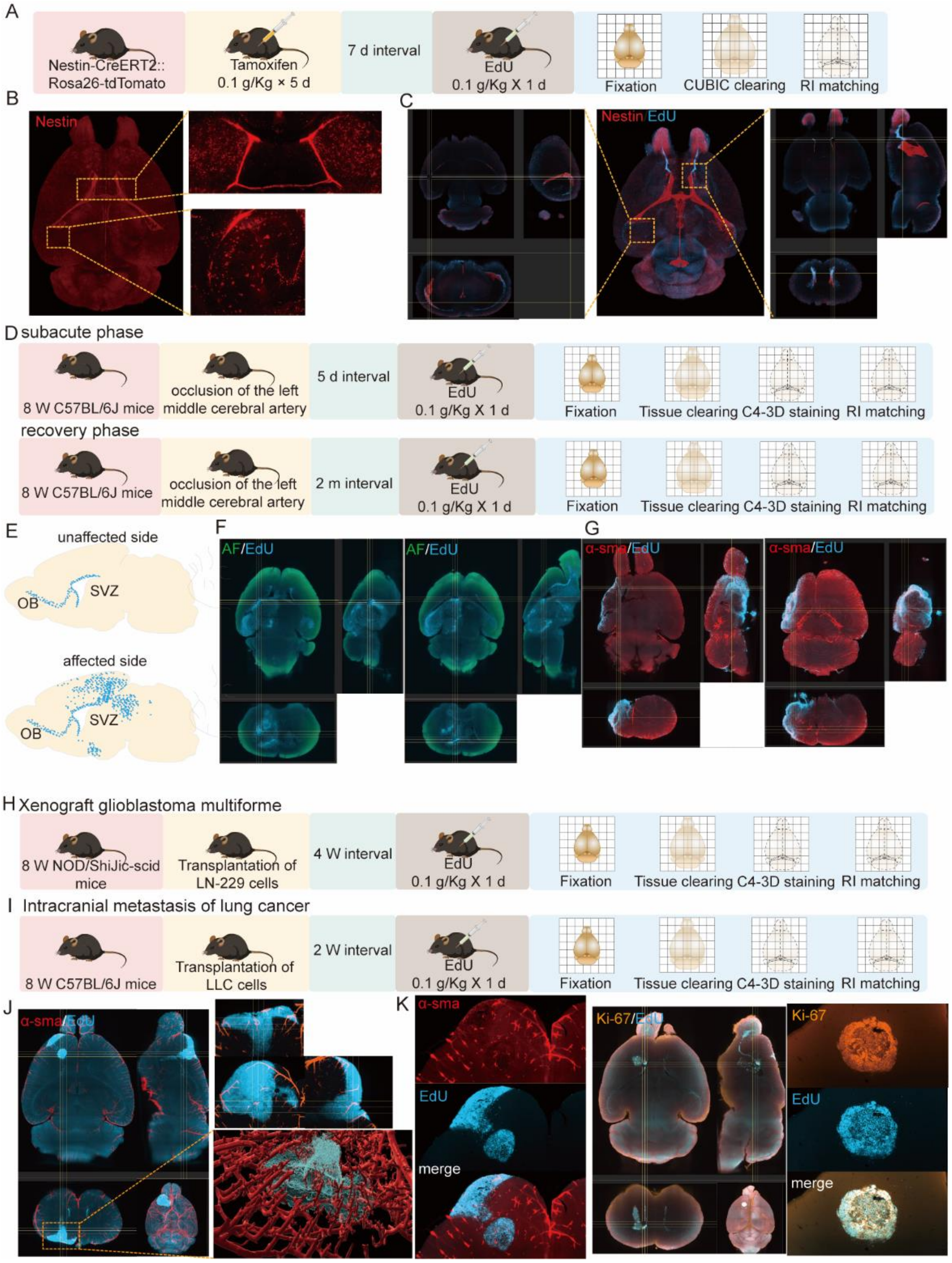
A
Nestin-CreERT2::
Rosa26-tdTomato
Tamoxifen
0.1 g/Kg × 5 d
7 d interval
EdU
0.1 g/Kg X 1 d
Fixation
CUBIC clearing
RI matching
B
Nestin
C
Nestin EdU
D subacute phase
8 W C57BL/6J mice
occlusion of the left middle cerebral artery
5 d interval
EdU
0.1 g/Kg X 1 d
Fixation
Tissue clearing
C4-3D staining
RI matching
recovery phase
8 W C57BL/6J mice
occlusion of the left middle cerebral artery
2 m interval
EdU
0.1 g/Kg X 1 d
Fixation
Tissue clearing
C4-3D staining
RI matching
E
unaffected side
OB
SVZ
affected side
OB
SVZ
F
AF/EdU
AF/EdU
G
α-sma/EdU
α-sma/EdU
H Xenograft glioblastoma multiforme
8 W NOD/ShiJic-scid mice
Transplantation of LN-229 cells
4 W interval
EdU
0.1 g/Kg X 1 d
Fixation
Tissue clearing
C4-3D staining
RI matching
I Intracranial metastasis of lung cancer
8 W C57BL/6J mice
Transplantation of LLC cells
2 W interval
EdU
0.1 g/Kg X 1 d
Fixation
Tissue clearing
C4-3D staining
RI matching
J
α-sma/EdU
K
α-sma
EdU
merge
Ki-67/EdU
Ki-67
EdU
merge

Figure S6. Application of C4-3D across diverse mouse models, including nestin reporter mouse expressing tdTomato (A–C, related to Figure 6), cerebral infarction and glioblastoma xenograft (D–K, related to Figure 7).

A Experimental procedures to visualize nestin and EdU signals in the nestin-Cre ERT2::Rosa26-tdTomato mouse brain at 8 weeks of age.

B Nestin signals (red) in 3D (left panel) and 2D (right panels).

C Nestin (red) and EdU (blue) signals were merged in 3D (left panel) and 2D (right panels).

D Schematic workflow for applying C^4-3D to a mouse model of cerebral infarction. Left middle cerebral artery occlusion (MCAO) was performed in 8-week-old C57BL/6J mice under isoflurane anesthesia. A silicone-coated filament was inserted via the external carotid artery to occlude the origin of the left middle cerebral artery. After 5 days for the analysis of subacute phase or 2 months for the analysis of recovery phase. Mice were sacrificed at the subacute phase (5 days post-occlusion) and recovery phase (2 months post-occlusion). EdU (0.1 g/kg) was administered 1 day prior to tissue collection. Brains were then processed through fixation, tissue clearing, C^4-3D staining, and refractive index (RI) matching for whole-brain 3D imaging.

E Schematic showing presumptive propagation of proliferating neuronal progenitors from the subventricular zone (SVZ) to the infarction site.

F, G EdU (blue), α-sma (red), and autofluorescence (AF, green) at five days **(F)** and two months **(G)** after left middle cerebral artery occlusion (MCAO). An identical region is indicated by three parallel lines in three different views.

H Schematic workflow for applying C^4-3D to a mouse model of xenograft glioblastoma multiforme (GBM). LN-229 human glioblastoma cells were stereotactically implanted into the striatum of 8-week-old NOD/ShiJic-scid mice under isoflurane anesthesia. After four weeks, EdU (0.1 g/kg) was intraperitoneally injected. On the next day, the brains were subjected to fixation, tissue clearing, C^4-3D staining, and refractive index (RI) matching for 3D imaging.

I Schematic workflow for applying C^4-3D to a mouse model of intracranial metastasis of lung cancer. Lewis lung carcinoma (LLC) cells were intravenously injected into 8-week-old C57BL/6J mice. After two weeks, EdU (0.1 g/kg) was intraperitoneally injected. On the next day, the brains were subjected to the C^4-3D pipeline as in panel **(H)**.

J, K EdU (blue) with α-sma (red) **(J)** and Ki-67 (orange) **(K)** of whole mouse brain transplanted with LN229 human glioblastoma cells.

Video S1. Representative image of tyrosine hydroxylase (red) and EdU (blue) of the mouse brain tissue-cleared by CUBIC. The video was generated by Imaris software, **related to Figure 1.**

Video S2. Representative automated identification of nuclear centers (red) from EdU signals (white) in the cortex, subventricular zone, dentate gyrus, glioblastoma, and stroke regions of the mouse brain tissue-cleared by CUBIC, **related to Figure 4.**

Video S3. Representative 3D image of EdU (blue) and Ki-67 (yellow) signals in the mouse brain tissue-cleared by iDisco+. Green signals represent autofluorescence. After 15 sec, Ki-67+/EdU+ nuclei are marked in green, and Ki-67+/EdU- nuclei are marked in red, **related to Figure 6.**

Video S4. Representative 3D image of EdU (blue) and nestin (red) signals in the mouse brain tissue-cleared by CUBIC. Green signals represent autofluorescence, **related to Figure 7.**

Video S5. Representative 3D image of EdU (blue) and α-smooth muscle actin (α-SMA, red) in the mouse brain two months after occlusion of the left middle cerebral artery. Note that EdU signals extend from the SVZ to the cortex, which is consistent with an established notion, **related to Figure 8.**

Videos S6, 7 and 8. Representative application of C^4-3D to visualize EdU signals (blue) in the kidney (Video S6), the liver (Video S7), and the lung (Video S8) of 8-week-old mice. Green signals represent autofluorescence, **related to Figure 9.**

Videos S1 to S8 are available at https://doi.org/10.6084/m9.figshare.29814998.v1.

Data S1. This dataset provides a manually annotated version of the Allen Brain Atlas, focusing on the subventricular zone (SVZ) in the caudoputamen subregion of the striatum. The SVZ is not included in the original Allen Brain Atlas, likely due to its thin structure consisting of only a few cell layers adjacent to the lateral ventricles. To address this omission, we manually segmented the SVZ by referencing previously reported definitions of the mouse SVZ. Using 2D slices of the anatomical atlas (average template.nrrd), a single layer of pixels was annotated on the lateral wall of the caudate nucleus region. The annotated voxels were assigned ID = 5 and incorporated into the atlas using a JSON editor, related to **Figure 6B**.

Data S2. This dataset provides a customized version of the Allen Brain Atlas, focusing on nestin-positive signals to delineate neuronal progenitor migration pathways. Using Nestin-Cre/ERT2-Rosa26-lsl-tdTomato mice treated with tamoxifen (200 mg/kg intraperitoneally for five consecutive days), nestin expression was visualized following tissue clearing with CUBIC and the application of C^4-3D. Coordinates of nestin-positive voxels were normalized to the Allen Brain Atlas. Voxels identified as nestin-positive in at least four out of seven mouse brains were annotated with ID = 5. This nestin-annotated atlas highlights the gradual decrease in nestin expression along migration pathways to the olfactory bulbs, enabling detailed spatial analysis, related to **Figure 6C**.

Data S1 and **Data S2** are available at https://doi.org/10.6084/m9.figshare.29815871.

References

1. Ollion, J., Cochennec, J., Loll, F., Escudé, C., and Boudier, T. (2013). TANGO: a generic tool for high-throughput 3D image analysis for studying nuclear organization. Bioinformatics *29*, 1840-1841. 10.1093/bioinformatics/btt276.
